# Polymorphism in the aggressive mimicry lure of the parasitic freshwater mussel *Lampsilis fasciola*

**DOI:** 10.7717/peerj.17359

**Published:** 2024-05-24

**Authors:** Trevor L. Hewitt, Paul D. Johnson, Michael Buntin, Talia Y. Moore, Diarmaid Ó Foighil

**Affiliations:** 1 Ecology and Evolutionary Biology, University of Michigan—Ann Arbor, Ann Arbor, Michigan, United States; 2 Alabama Aquatic Biodiversity Center, Marion, Alabama, United States of America; 3 Robotics Department & Mechanical Engineering Department, University of Michigan—Ann Arbor, Ann Arbor, Michigan, United States

**Keywords:** Mimicry, Freshwater mussels, ddRAD, Behavior, Parasitism, Polymorphism, Captive brood

## Abstract

Unionoid freshwater mussels (Bivalvia: Unionidae) are free-living apart from a brief, obligately parasitic, larval stage that infects fish hosts, and gravid female mussels have evolved a spectrum of strategies to infect fish hosts with their larvae. In many North American species, this involves displaying a mantle lure: a pigmented fleshy extension that acts as an aggressive mimic of a host fish prey, thereby eliciting a feeding response that results in host infection. The mantle lure of *Lampsilis fasciola* is of particular interest because it is apparently polymorphic, with two distinct primary lure phenotypes. One, described as “darter-like”, has “eyespots”, a mottled body coloration, prominent marginal extensions, and a distinct “tail”. The other, described as “worm-like”, lacks those features and has an orange and black coloration. We investigated this phenomenon using genomics, captive rearing, biogeographic, and behavioral analyses. Within-brood lure variation and within-population phylogenomic (ddRAD-seq) analyses of individuals bearing different lures confirmed that this phenomenon is a true polymorphism. The relative abundance of the two morphs appears stable over ecological timeframes: the ratio of the two lure phenotypes in a River Raisin (MI) population in 2017 was consistent with that of museum samples collected at the same site six decades earlier. Within the River Raisin, four main “darter-like” lure motifs visually approximated four co-occurring darter species (*Etheostoma blennioides, E. exile, E. microperca*, and *Percina maculata*), and the “worm-like” lure resembled a widespread common leech, *Macrobdella decora*. Darters and leeches are typical prey of *Micropterus dolomieui* (smallmouth bass), the primary fish host of *L. fasciola*. *In situ* field recordings of the *L. fasciola* “darter” and “leech” lure display behaviors, and the lure display of co-occurring congener *L. cardium*, were captured. Despite having putative models in distinct phyla, both *L. fasciola* lure morphs have largely similar display behaviors that differ significantly from that of sympatric *L. cardium* individuals. Some minor differences in the behavior between the two *L. fasciola* morphs were observed, but we found no clear evidence for a behavioral component of the polymorphism given the criteria measured. Discovery of discrete within-brood inheritance of the lure polymorphism implies potential control by a single genetic locus and identifies *L. fasciola* as a promising study system to identify regulatory genes controlling a key adaptive trait of freshwater mussels.

## Introduction

In ecology, mimicry refers to a convergent adaptive trait prevalent in many biological communities: the deceptive resemblance of one organism to another (Pasteur, 1982; Schaefer & Ruxton, 2009; Maran, 2015). It involves three categories of interacting ecological players: mimic (organism displaying the deceptive resemblance), model (organism being mimicked), and receiver (organism being deceived) (Pasteur, 1982; Maran, 2015). Mimicry occurs across a wide variety of ecological contexts and sensory modalities, but conceptually (Jamie, 2017), individual cases can be categorized by the traits being mimicked (signals or cues), as well as by the degree of deceptiveness (aggressive, rewarding, Müllerian or Batesian mimicry). Mimicry is also ubiquitous throughout nature, with many prominent well studied examples including mantids (O’Hanlon, Holwell & Herberstein, 2014), spiders (Ceccarelli, 2013), fish (Randall, 2005), and many more.

Mimetic systems that are polymorphic (multiple within-species mimic morphs with discrete models) have been particularly influential in uncovering the genetic basis of complex adaptive traits in natural populations (Clarke, Sheppard & Thornton, 1968; Jay et al., 2018; Palmer & Kronforst, 2020). Such polymorphisms are rare in nature, with the most well studied examples occurring in papilionid butterflies (Clarke, Sheppard & Thornton, 1968; Clarke & Sheppard, 1971; Hazel, 1990; Joron & Mallet, 1998; Nijhout, 2003). For instance, polymorphisms in *Heliconious* species are determined by presence/absence of an introgressed chromosomal inversion ‘supergene’ (Jay et al., 2018), and alleles of a single ancestral gene (*doublesex*) control female-specific polymorphisms in *Papilio* species (Palmer & Kronforst, 2020).

**Figure 1:**
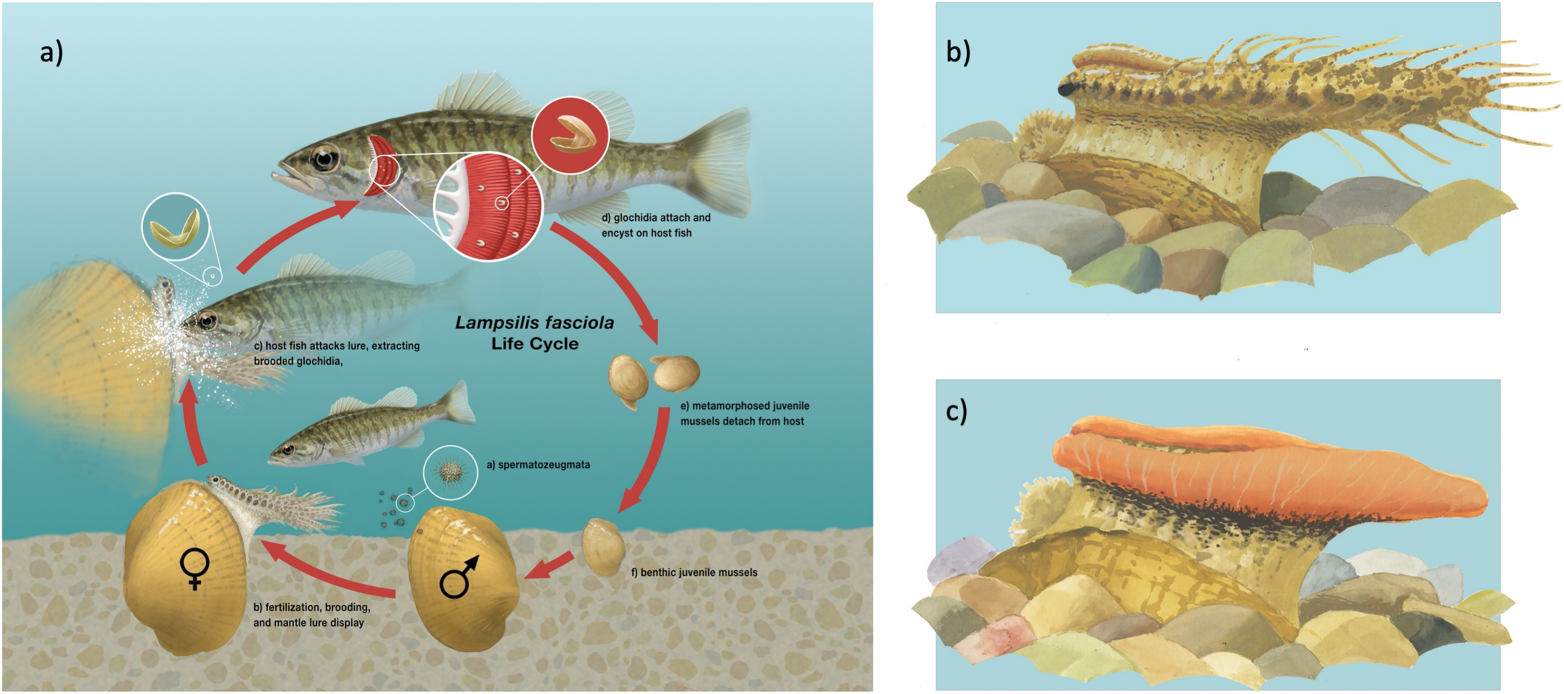
Illustration of *Lampsilis fasciola* life cycle. (A) The life cycle of the freshwater mussel *Lampsilis fasciola*. A gravid female mussel displays a mantle lure, here a darter mimic, to the primary fish host, *Micropterus dolomieu*. This elicits an attack through which the host is infected by mussel parasitic larvae (glochidia). After a short infective period (~2 weeks), the parasitic larvae metamorphose into juvenile mussels that detach from the host and fall to the substrate. (B) (“darter-like”) and (C) (“worm-like”) depict the two primary phenotypes of lure observed in *L. fasciola*. The former (B) has “eyespots”, a mottled “main body” pigmentation composed of lateral and dorsal spots that can vary substantially in color, numerous and prominent marginal extensions, and a distinct “tail” region, whereas the latter lacks those features and has instead a uniform bright orange coloration underlain with a black basal stripe. Illustration by John Megahan.

In contrast to papilionid butterflies, the genetics of mimicry trait evolution among unionoid mussels is poorly understood. Unionoida comprise ~75% of the planet’s freshwater bivalve species and are free-living apart from a brief, obligately parasitic, larval stage that infects fish hosts (Bogan, 2007; Haag, 2012). Gravid female mussels have evolved a spectrum of strategies to infect hosts with their larvae (Zanatta & Murphy, 2006; Barnhart, Haag & Roston, 2008; Hewitt, Wood & Ó Foighil, 2019). Females in many species use a mantle lure (Welsh, 1933): a pigmented fleshy extension that provides a visual cue resembling the prey of host fish, eliciting a feeding response that results in host infection (Haag & Warren, 1999; Barnhart, Haag & Roston, 2008; Fig. 1A). Many species also have a behavioral component; usually in the form of lateral undulations that travel as a wave along the edges of each half (right and left) of the mantle lure (Ortmann, 1921; Barnhart, Haag & Roston, 2008). Although this behavior was observed and described in the early 20^th^ century (Ortmann, 1921), it was not until much later that Haag & Warren (1999) observed how this behavior was used to attract strikes from host fish. The mantle lure presents itself as a reward to potential host fish but is deceptive in nature and leads to parasitization of the host fish. This mimetic system can therefore be classified as an example of aggressive mimicry following the definition by Jamie (2017). The variability in lure display behavior among species of unionid is not well understood. Mimetic mantle lures predominate in Lampsilini, a major clade of North American freshwater mussels recently identified as a cryptic adaptive radiation centered on larval ecologies and specialized host-infection behaviors (Hewitt, Haponski & Ó Foighil, 2021b). This interaction is referred to as ‘cryptic’ because the specific host-parasite interactions are transient and difficult to determine *in-situ*. Ortmann (1921) and Kramer (1970) reported the production of rudimentary mantle lures in juveniles and male lampsilines, but noted that formation of fully developed lures is restricted to sexually mature females, and that only gravid females engage in lure display behaviors. Surprisingly, neither Ortmann (1921) nor Kramer (1970) depicted male mussel lure rudiments, nor could we find any such depictions in the literature.

Although mimetic mantle lures are a key adaptive trait of freshwater mussel diversification, the genetic regulators underlying their formation (Kramer, 1970), variation (Haag, Warren & Shillingsford, 1999; Zanatta, Fraley & Murphy, 2007; Barnhart, Haag & Roston, 2008), and evolution (Zanatta & Murphy, 2006; Hewitt, Haponski & Ó Foighil, 2021b) remain completely unknown. This gap in our knowledge is exacerbated by the stark conservation status of North American freshwater mussels, with two thirds of species classified as threatened or near-threatened (Lopes-Lima et al., 2018).

As with papilionid butterflies (Jay et al., 2018; Palmer & Kronforst, 2020), targeting polymorphic lampsiline mantle lures for in-depth study may represent a tractable route to closing that gap between genes and phenotypes. *Lampsilis fasciola*, the Wavy-Rayed Lampmussel, is a promising candidate species in that it produces a number of distinct mantle lure phenotypes (Zanatta, Fraley & Murphy, 2007) across its Eastern North America distribution, extending from southern Ontario to northern Alabama (Parmalee & Bogan, 1998). Two range-wide lure phenotypes predominate in northern populations. The more common of the two, labeled “darter-like” by Zanatta, Fraley & Murphy (2007), has “eyespots”, a mottled “main body” pigmentation composed of lateral and dorsal spots that can vary substantially in color, numerous and prominent marginal extensions (AKA “appendages” or “tentacles”), and a distinct “tail” region (Kramer, 1970; Zanatta, Fraley & Murphy, 2007; Fig. 1B). A rarer lure phenotype, labeled “worm-like” by McNichols (2007), lacks the above features and has instead a uniform bright orange coloration underlain with a black basal stripe (Zanatta, Fraley & Murphy, 2007; Fig. 1C). The latter lure phenotype is highly distinctive within the genus *Lampsilis* where fish-like mantle lures are the norm (Kramer, 1970). Much work has been done in attempt to quantify similarity between models and mimics, and qualitatively assess most likely models (Kelly et al., 2021), but defining models for lampsiline lure mimics thus far has largely been based on visual similarities defined by expert opinion (Zanatta, Fraley & Murphy, 2007; Barnhart, Haag & Roston, 2008). Based on the results of laboratory larval infection experiments and on the degree of ecological overlap, *Micropterus dolomieu* (Smallmouth Bass), and to a lesser extent *Micropterus salmoides* (Largemouth Bass), have been identified as *L. fasciola’s* primary fish hosts (Zale & Neves, 1982; McNichols, 2007; Morris et al., 2008; McNichols, Mackie & Ackerman, 2011; VanTassel et al., 2021). Both host species are generalist predators of aquatic invertebrates and vertebrates (Clady, 1974).

Our study aimed to address outstanding, interrelated questions to develop *L. fasciola* into an integrated mantle lure polymorphism study system. First among them was residual uncertainty that the mantle lure morphs represent polymorphisms rather than cryptic species. Zanatta, Fraley & Murphy (2007), using microsatellite markers, did not detect evidence of cryptic species but qualified their conclusions due to small sample sizes, and their result requires corroboration (Fisheries & Oceans Canada, 2018). Secondly, we currently lack any data on the mantle lure phenotype ratios over time (or on a mechanism for its presumed maintenance). Thirdly, we attempt to define respective models of each *L. fasciola* mantle lure mimic in a natural population. Finally, mantle lure display behavior is an important component of effective mimicry in freshwater mussels (Welsh, 1933; Jansen, Bauer & Zahner-Meike, 2001; Haag & Warren, 2003; Barnhart, Haag & Roston, 2008), but it is unknown if morphologically divergent *L. fasciola* mantle lures, that presumably mimic very distinct host prey models, also differ in their display behaviors. We tested this by making and analyzing video recordings of lure movements of displaying polymorphic females in a natural population over 3 years. We used a combination of field-collection, captive breeding, museum specimens, and ecological surveys to collect genetic, phenotypic, and population data on this species. This publication was first released as a preprint (Hewitt et al., 2023; doi: https://doi.org/10.1101/2023.11.27.568842), however, the version presented here is the official peer-reviewed publication.

## Materials and Methods

### Tissue sample collection

**Figure 2:**
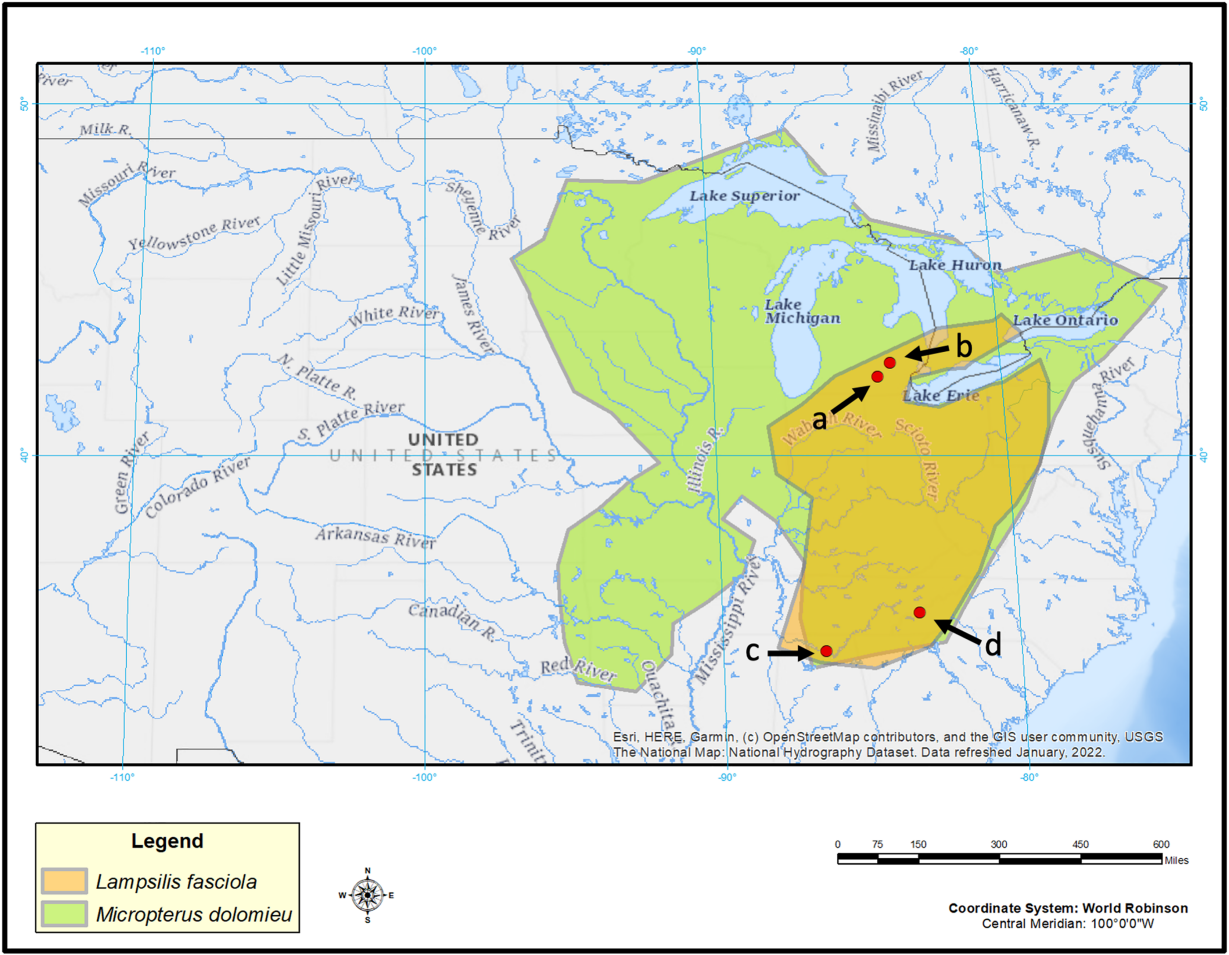
Map displaying geographic range of *Lampsilis fasciola* and its primary host, *Micropterus dolomieu*, as well as sampling locations. Map of eastern North America showing the estimated ranges of *Lampsilis fasciola* (orange) and of its primary host fish *Micropterus dolomieu* (green). Red dots indicate sampling sites: Raisin River at Sharon Mils County Park (A), Huron River at Hudson Mills Park (B), Paint Rock River (C) and Little Tennessee River (D). Base map layer is from U.S. Geological Survey (2022).

*L. fasciola* mantle tissue samples were collected for genotyping purposes by taking non-lethal mantle clip biopsies (Berg et al., 1995) from wild population lure-displaying female mussels during the summers of 2017, 2018, and 2021 in three rivers (Fig. 2). Maps were made in ArcGIS (ESRI, 2022) using U.S. Geological Survey (2022) as a basemap layer. Two of the sampling locations were in southeastern Michigan: the River Raisin at Sharon Mills County Park (42.176723, −84.092453; *N* = 30; 24 “darter-like”, six “worm-like”, collectively sampled in 2017, 2018 & 2020), and the Huron River at Hudson Mills Metropark, MI (42.37552, −83.91650; *N* = 13; 7 “darter-like”, six “worm-like”, collectively sampled in 2017, 2018, and 2020 under the MI Threatened and endangered species collection permit TE149). Both rivers flow into Lake Erie and are part of the Saint Lawrence drainage. The third location was in North Carolina: the Little Tennessee River (N = 10; 35.32324, −83.52275; *N* = 10, all were “darter-like” and sampled in 2017); this river is a tributary of the Tennessee River and part of the Mississippi drainage. Prior to each biopsy, photographs of the intact, undisturbed, lure display were taken with an Olympus Tough TG-6 underwater camera (Fig. S1).

### Captive brood tissue samples

We also obtained tissue samples from 50 captive-raised individuals of a single brood that had been ethanol-preserved. In 2009, the Alabama Aquatic Biodiversity Center (AABC) established a culture facility for endangered freshwater mussels. The Center’s inaugural culture attempt, by co-authors Paul Johnson and Michael Buntin, was a proof-of-concept trial involving a single gravid female *L. fasciola* sourced from the Paint Rock River (another Tennessee River tributary; *N* 34˚ 47.733′,W 86˚ 14.396′) in Jackson County, AL (Fig. 2) on June 11, 2009. This female *L. fasciola* had a “worm-like” lure: the AABC data sheet for the trial 2009 host infection (Fig. S2) records that it was “bright orange and black” and lacked the “eyespots”, mottled body coloration, marginal extensions, and “tail” of the “darter-like” lure phenotype (Buntin & Johnson, 2009, personal observations). On July 13 2009, about 31,000 glochidia larvae were extracted from the female’s marsupia and used to infect *Micropterus coosae* (Redeye Bass) hosts sourced from the Eastaboga Fish Hatchery (Calhoun County, AL, USA) using standard protocols (Barnhart, Haag & Roston, 2008). The female mussel was then returned live to the Paint Rock River. Following completion of larval development on the fish hosts, about 9,300 metamorphosed juvenile mussels were recovered and reared, initially for the first few weeks in mucket bucket systems (Barnhart, 2006), then in a suspended upwelling system (SUPSYS) for 2 years with about 2,200 surviving. In 2011, this proof-of-concept culture experiment was terminated, and the survivors were donated to several research groups, with the majority used for toxicology experiments (Leonard et al., 2014a, 2014b).

**Figure 3:**
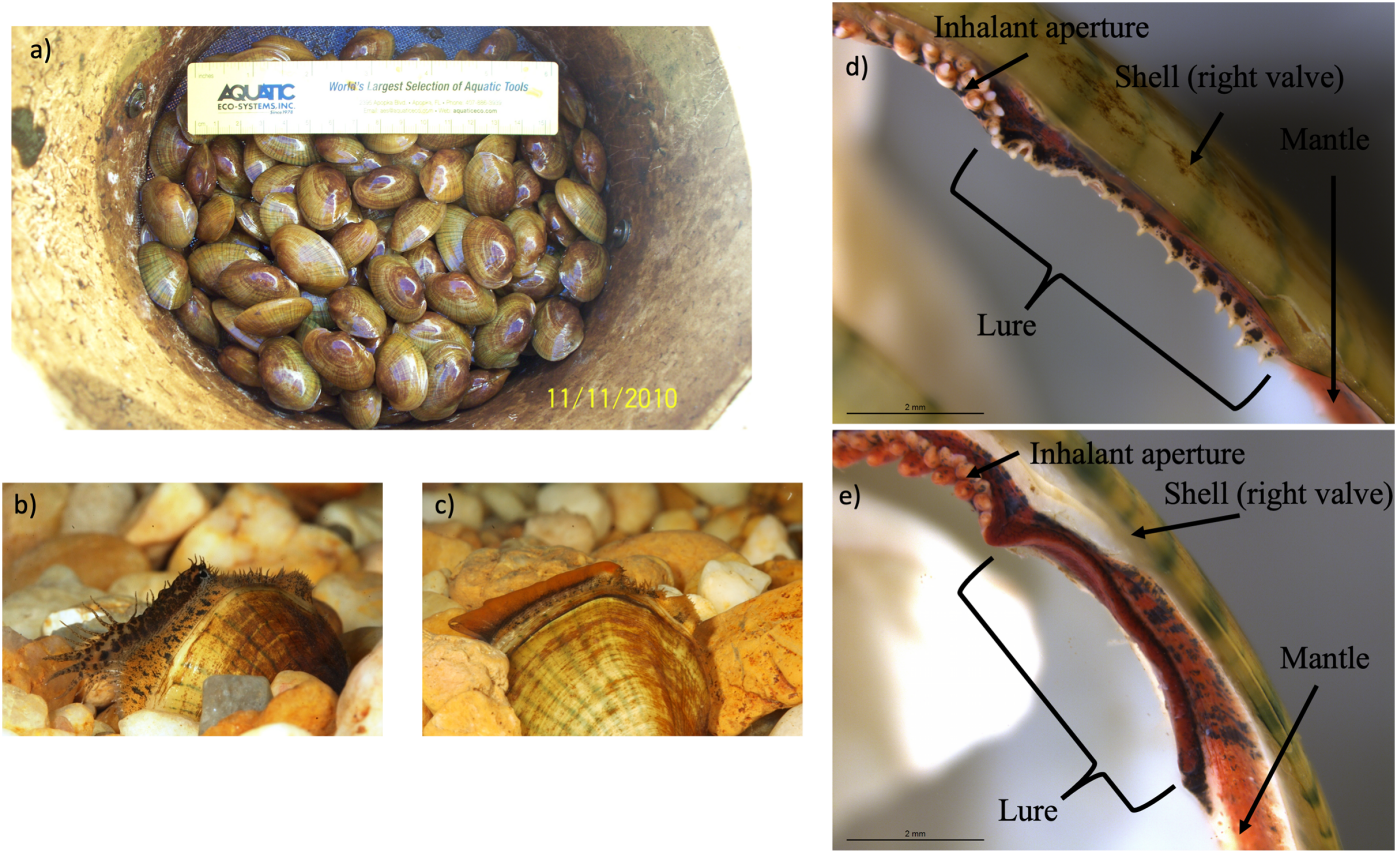
Photographs of *Lampsilis fasciola* brood raised at the Alabama Aquatic Biodiversity Center, as well as photographs of preserved lure rudiments. The *Lampsilis fasciola* brood raised at the Alabama Aquatic Biodiversity Center from a wild, gravid female, with a “worm-like” mantle lure, sampled from the Paint Rock River in June 2009. (A) shows juvenile members of the brood after ~16 months in culture. (B and C) show single, sexually maturing females after ~2 years of culture. The young female in (B) displayed a developing “darter-like” mantle lure (with “eyespots”, mottled lateral coloration, marginal extensions, and a “tail”) whereas her full- or half-sibling in (C) displayed a “worm-like” mantle lure (lacking the “darter” characteristics and having orange pigmentation with a black underlay). (D and E) Respectively show photomicrographs, taken with a dissecting microscope, of 95% ethanol-preserved rudimentary “darter-like” and “worm-like” lures from two additional brood members, part of a 50-individual subsample preserved in 2011.

Prior to the brood’s termination, Johnson noticed that a few females had attained sexual maturity and were displaying polymorphic lures (Figs. 3B, 3C). To substantiate that 2011 observation, we examined 50 individuals that had been preserved in 95% ethanol and shipped to Nathan Johnson (USGS) in Gainsville, FL in 2011. Because *Lampsilis* spp. juveniles and males produce a rudimentary mantle lure (Ortmann, 1921; Kramer, 1970), we were able to determine the primary lure phenotype (darter-like” or “worm-like”) of all 50 preserved brood members. Using a Leica MZ16 dissecting microscope, individual photomicrographs were taken of the preserved rudimentary lure structures (Figs. 3D, 3E and S3), and their respective lure phenotypes were identified independently by both T. Hewitt and by D. Ó Foighil. Additionally, tissue samples were acquired from all 50 individuals and included for phylogenomic analyses.

### Phylogenomic analyses

DNA sequencing and raw data processing were performed using the protocol outlined in Hewitt, Haponski & Ó Foighil (2021a, 2021b). Genomic DNA was extracted from tissue samples using E.Z.N.A. Mollusk DNA kit (Omega Bio-Tek, Norcross, GA, USA) according to manufacturer’s instructions and then stored at −80 °C. The quality and quantity of DNA extractions were assessed using a Qubit 2.0 Fluorometer (Life Technologies, Carlsbad, CA, USA) and ddRADseq libraries were prepared following the protocols of Peterson et al. (2012). We then used 200 ng of DNA for each library prep. This involved digestion with Eco-RI-HF and MseI (New England Biolabs, Ipswich, MA, USA) restriction enzymes, followed by isolating 294–394 bp fragments using a Pippen Prep (Sage Science, Beverly, MA, USA) following the manufacturer’s instructions. Prepared ddRADseq libraries then were submitted to the University of Michigan’s DNA sequencing core and run in four different lanes using 150 bp paired-end sequencing on either an Illumina HiSeq 2500 or Illumina novaseq shared flow cell. Two control individuals of *L. fasciola* were run in each lane and reads for both individuals clustered together in every analysis with 100% bootstrap support, indicating no lane effects on clustering among individuals. Raw demultiplexed data were deposited at GenBank under the bioproject ID PRJNA985631 with accession numbers SAMN35800743–SAMN35800847. Individuals included in phylogenomic analyses can be found in Table 1, and museum ID numbers can be found in Table S1.

**Table 1:** The name, phenotype, and sequencing metrics. Raw reads, total clusters, and total loci in assembly from the ddRAD sequencing are displayed for each genotyped sample of Lampsilis fasciola and of the outgroup taxa. Individual *Lampsilis fasciola* lure phenotype designation followed (Zanatta, Fraley & Murphy, 2007). Museum ID numbers can be found in Table S1.

Sample name	Lure phenotype	Raw reads	Total clusters	Average clustering depth	Loci in assembly
L_fasciola_AL_brood_1	Worm-like	258,664	97,681	2.14	483
L_fasciola_AL_brood_2	Darter-like	5,201,836	1,120,710	3.28	25,686
L_fasciola_AL_brood_3	Worm-like	5,492,519	1,126,749	3.4	25,703
L_fasciola_AL_brood_4	Darter-like	2,429,494	632,254	2.84	21,398
L_fasciola_AL_brood_5	Worm-like	3,152,003	760,260	3.02	23,761
L_fasciola_AL_brood_6	Darter-like	3,212,851	810,898	2.87	23,434
L_fasciola_AL_brood_7	Darter-like	3,649,891	593,765	4.22	25363
L_fasciola_AL_brood_8	Darter-like	4,869,307	1,462,723	2.29	19,089
L_fasciola_AL_brood_9	Worm-like	3,158,818	718,169	3.08	23,033
L_fasciola_AL_brood_10	Darter-like	4,000,321	915,881	3.12	24,916
L_fasciola_AL_brood_11	Worm-like	5,679,854	1,171,842	3.35	25,770
L_fasciola_AL_brood_12	Darter-like	4,212,783	979,265	3.04	24,693
L_fasciola_AL_brood_13	Worm-like	1,300,563	399,134	2.51	12,145
L_fasciola_AL_brood_14	Darter-like	4,100,372	1,043,360	2.79	23,521
L_fasciola_AL_brood_15	Darter-like	5,804,293	1,412,102	2.91	25,570
L_fasciola_AL_brood_16	Worm-like	1,555,906	427,061	2.7	14,099
L_fasciola_AL_brood_17	Darter-like	2,073,968	598,680	2.59	13,668
L_fasciola_AL_brood_18	Worm-like	6,919,783	1,574,429	3.08	25,811
L_fasciola_AL_brood_19	Darter-like	3,434,210	829,507	2.94	23,708
L_fasciola_AL_brood_20	Darter-like	4,778,853	994,416	3.35	25,500
L_fasciola_AL_brood_21	Worm-like	2,462,560	590,095	2.91	20,588
L_fasciola_AL_brood_22	Worm-like	6,600,876	1,406,451	3.26	26,080
L_fasciola_AL_brood_23	Darter-like	7,090,859	1,628,965	3.06	25,932
L_fasciola_AL_brood_24	Worm-like	4,546,435	1,061,394	3	24,174
L_fasciola_AL_brood_25	Worm-like	5,379,577	1,135,906	3.35	25,703
L_fasciola_AL_brood_26	Worm-like	5,592,652	1,501,130	2.67	23,965
L_fasciola_AL_brood_27	Worm-like	4,893,957	825,855	4.09	25,924
L_fasciola_AL_brood_28	Darter-like	2,596,873	519,103	3.59	22,103
L_fasciola_AL_brood_29	Darter-like	3,401,334	883,485	2.87	21,377
L_fasciola_AL_brood_30	Worm-like	3,876,395	1,014,133	2.8	22,072
L_fasciola_AL_brood_31	Worm-like	5,391,442	1,246,528	3.07	25,009
L_fasciola_AL_brood_32	Darter-like	4,365,005	1,084,596	2.85	23,030
L_fasciola_AL_brood_33	Darter-like	5,116,507	1,117,916	3.16	24,667
L_fasciola_AL_brood_34	Darter-like	7,480,755	1,601,100	3.19	26,163
L_fasciola_AL_brood_35	Darter-like	8,121,426	1,825,135	3.02	25,972
L_fasciola_AL_brood_36	Darter-like	5,521,997	1,414,238	2.78	24,163
L_fasciola_AL_brood_37	Darter-like	6,562,641	1,579,514	2.88	25,476
L_fasciola_AL_brood_38	Darter-like	6,303,766	1,596,624	2.76	24,448
L_fasciola_AL_brood_39	Darter-like	6,206,795	1,488,925	2.91	24,648
L_fasciola_AL_brood_40	Darter-like	8,630,897	1,891,164	3.11	26,176
L_fasciola_AL_brood_41	Darter-like	7,293,683	1,716,571	2.95	25,604
L_fasciola_AL_brood_42	Darter-like	4,896,252	1,193,262	2.88	22,829
L_fasciola_AL_brood_43	Darter-like	6,098,052	1,471,714	2.9	25,074
L_fasciola_AL_brood_44	Darter-like	7,495,994	1,698,871	3.04	25,701
L_fasciola_AL_brood_45	Darter-like	3,937,758	670,698	4.06	24,947
L_fasciola_AL_brood_46	Darter-like	6,370,942	1,343,655	3.26	25,855
L_fasciola_AL_brood_47	Darter-like	5,542,864	1,318,463	2.96	24,550
L_fasciola_AL_brood_48	Darter-like	6,313,913	1,469,606	2.98	24,983
L_fasciola_AL_brood_49	Darter-like	3,163,000	789,239	2.9	24,776
L_fasciola_AL_brood_50	Darter-like	1,728,370	548,529	2.35	17,837
L_fasciola_Huron_5	Darter-like	953,302	259,898	2.8	10,996
L_fasciola_Huron_6	Worm-like	1,682,931	362,706	3.31	16,809
L_fasciola_Huron_7	Worm-like	746,944	157,212	3.29	10,644
L_fasciola_Huron_8	Worm-like	1,899,689	402,515	3.25	16,584
L_fasciola_Huron_9	Darter-like	1,213,655	293,090	2.97	11,818
L_fasciola_Huron_10	Darter-like	7,775,910	1,275,602	3.87	22,035
L_fasciola_Huron_11	Darter-like	1,533,281	295,767	3.55	15,386
L_fasciola_NC_1	Darter-like	1,308,813	254,002	3.61	11,873
L_fasciola_NC_2	Darter-like	4,862,573	852,380	3.77	18,321
L_fasciola_NC_3	Darter-like	663,874	165,869	2.95	9,960
L_fasciola_NC_4	Darter-like	2,610,453	465,228	3.76	13,790
L_fasciola_NC_5	Darter-like	6,927,947	1,459,334	3.05	20,804
L_fasciola_NC_6	Darter-like	1,051,195	202,171	3.27	12,415
L_fasciola_NC_7	Darter-like	1,948,092	382,878	3.61	17,101
L_fasciola_NC_8	Darter-like	3,475,751	669,278	3.69	20,683
L_fasciola_NC_9	Darter-like	5,693,936	1,634,946	2.46	22,325
L_fasciola_NC_10	Darter-like	2,175,381	464,794	3.38	17,094
L_fasciola_NC_11	Darter-like	2,189,933	516,643	3.05	17,580
L_fasciola_Redo_1	Darter-like	1,455,864	327,622	2.62	13,478
L_fasciola_Redo_2	Darter-like	1,839,020	436,418	2.43	13,181
L_fasciola_Raisin_2	Darter-like	8,235,827	1,716,137	3.29	25,555
L_fasciola_Raisin_3	Darter-like	6,032,935	1,488,448	2.85	25,006
L_fasciola_Raisin_4	Darter-like	12,947,164	3,587,458	2.45	25,245
L_fasciola_Raisin_1	Darter-like	6,639,384	1,086,218	3.97	23,458
L_fasciola_Raisin_5	Darter-like	10,059,843	1,997,619	3.41	25,363
L_fasciola_Raisin_6	Darter-like	8,019,689	1,847,955	3.01	25,769
L_fasciola_Raisin_7	Darter-like	3,816,242	681,697	3.95	24,606
L_fasciola_Raisin_8	Darter-like	6,117,037	1,282,299	3.27	22,439
L_fasciola_Raisin_9	Worm-like	5,170,380	775,979	4.64	25,798
L_fasciola_Raisin_10	Darter-like	761,451	176,858	3.14	11,477
L_fasciola_Raisin_11	Worm-like	7,140,657	1,670,143	2.97	25,519
L_fasciola_Raisin_12	Darter-like	890,521	203,114	2.91	10,582
L_fasciola_Raisin_13	Darter-like	1,071,361	225,030	3.47	13,512
L_fasciola_Raisin_14	Darter-like	3,644,379	946,273	2.82	21,995
L_fasciola_Raisin_15	Darter-like	3,578,043	482,446	5.04	17,514
L_fasciola_Raisin_16	Darter-like	2,351,544	114,072	14.25	516
L_fasciola_Raisin_17	Darter-like	5,272,816	1,304,726	2.87	23,305
L_fasciola_Huron_1	Worm-like	13,366,692	4,050,829	2.26	17,555
L_fasciola_Huron_2	Darter-like	2,819,896	928,226	2.24	20,205
L_fasciola_Huron_3	Darter-like	662,275	186,602	2.66	7,653
L_fasciola_Huron_4	Darter-like	4,792,093	855,457	3.88	24,512
L_fasciola_AL_mom_1	Darter-like	8,095,030	1,840,917	2.95	25,420
L_fasciola_AL_mom_2	Darter-like	10,329,331	3,504,027	2.03	24,488
L_fasciola_AL_mom_3	Darter-like	10,384,477	2,987,559	2.34	25,056
L_fasciola_Huron_12	Worm-like	6,906,349	1,672,394	2.87	25,281
L_fasciola_Huron_13	Worm-like	6,955,496	1,670,627	2.88	25,593
L_fasciola_Raisin_18	Worm-like	5,506,215	1,301,878	3	25,373
L_fasciola_Raisin_19	Worm-like	6,611,596	1,524,682	3.03	25,604
L_fasciola_Raisin_20	Worm-like	4,894,495	1,276,608	2.74	24,931
L_fasciola_Raisin_21	Worm-like	8,396,562	1,736,736	3.26	25,490
L_cardium_1		6,864,226	1,710,220	2.8	14,625
L_cardium_2		4,898,330	1,091,622	3.11	13,433
L_cardium_3		7,109,883	2,005,565	2.5	14,563
L_cardium_4		4,637,077	997,208	3.27	13,860
S_nasuta_1		4,544,989	1,169,260	2.55	10,441

The alignment-clustering algorithm in ipyrad v.0.7.17 (Eaton, 2014; Eaton & Overcast, 2020) was used to identify homologous ddRADseq tags. Ipyrad is capable of detecting insertions and deletions among homologous loci, which increases the number of loci recovered at deeper evolutionary scales compared to alternative methods of genomic clustering (Eaton, 2014). Demultiplexing was performed by sorting sequences by barcode, allowing for zero barcode mismatches (parameter 15 setting 0) and a maximum of five low-quality bases (parameter 9). Restriction sites, barcodes, and Illumina adapters were trimmed from the raw sequence reads (parameter 16 setting 2), and bases with low-quality scores (Phred-score < 20, parameter 10 setting 33) were replaced with an *N* designation. Sequences were discarded if they contained more than 5 N’s (parameter 19). Reads were clustered and aligned within each sample at an 85% similarity threshold, and clusters with a depth <6 were discarded (parameters 11 and 12). We also varied the number of individuals required to share a locus from ~50% to ~75%.

We analyzed the two concatenated ddRAD-seq alignment files (50% and 75% minimum samples per locus) using maximum likelihood in RAxML v8.2.8 (Stamatakis, 2014). A general time-reversible model (Lanave et al., 1984) was used for these analyses that included invariable sites and assumed a gamma distribution. Support was determined for each node using 100 fast parametric bootstrap replications. Lure phenotype information was recorded and mapped on to the phylogenetic tree. Phylogenetic signal of lure phenotype was tested using Pagel’s (1999) *λ* in R (R Core Team, 2018) with the ‘phylobase’ package (Hackathon et al., 2013).

### River raisin mantle lure phenotype ratios over time

Mid-20^th^ century *L. fasciola* specimens collected at the Sharon Mills County Park site (Raisin River, MI, USA; Fig. 2A) are preserved as part of the University of Michigan’s Museum of Zoology wet mollusk collection. They stem from eight different collecting events between 1954 and 1962 (Table S2), and their presence afforded an opportunity to assess the stability of the *L. fasciola* “darter/worm” mantle lure polymorphism in that population over a six-decade time interval. All of the museum specimens, males as well as females, were examined to determine whether their fully-formed (female) or rudimentary (male) mantle lures were “darter-like” or “worm-like”. For females, this could be achieved by simple visual examination, but male lure classification required a dissecting microscope. The percentages of mantle lure phenotypes observed in the Sharon Mills County Park population was compared among mid-20th century (UMMZ preserved females and males) and 2017 (field photographs and videos of displaying females) samples using a Fisher’s exact test, implemented in R.

### Putative lure mimicry models

Population-specific putative model species for the *L. fasciola* mantle lure mimicry system were investigated at the River Raisin Sharon Mills County Park study site (Fig. 2), in part because of the availability of a comprehensive ecological survey of Raisin River fishes (Smith, Taylor & Grimshaw, 1981). “Darters”—members of the speciose North American subfamily Etheosomatinae— have been implictly identified as models for the predominant “darter-like” mantle lure phenotype (Zanatta, Fraley & Murphy, 2007), and they are preyed upon by *Micropterus dolomieu* (Surber, 1941; Robertson & Winemiller, 2001; Murphy et al., 2005), *L. fasciola’s* primary fish host (Zale & Neves, 1982; McNichols, 2007; Morris et al., 2008; McNichols, Mackie & Ackerman, 2011; VanTassel et al., 2021). Ten species of Etheosomatinae occur in the River Raisin, as does *M. dolomieu* (Smith, Taylor & Grimshaw, 1981).

River Raisin gravid female *L. fasciola* engage in mantle lure displays from May-August. During the summer of 2017, a total of 27 different displaying females were photographed along a 150-m stretch downstream of the dam at Sharon Mills County Park using an Olympus Tough TG-6 underwater camera. Individuals were located by carefully scanning the river bed with mask and snorkel to try and approximate the real ratios of phenotypes at this site. Additional lure photos were taken by coauthor Paul Johnson at the AABC of individuals from the Paint Rock River (AL). The lures were first categorized into broad groupings based on visual similarity, in terms of morphology and coloration. These groupings were then used to identify putative host prey fish model species from those present in the River Raisin drainage (Smith, Taylor & Grimshaw, 1981), based on similarities in size, shape, and coloration. Putative model species were further assessed based on their relative local abundance (Smith, Taylor & Grimshaw, 1981) and on their range overlap with both mimic and receiver. We also photograph and document the male rudimentary lures for both *L. fasciola* and *L. cardium*, taken from the River Raisin (Fig. S4). Geographic ranges of *L. fasciola*, the primary host *M. dolomieu*, and each prospective model species were produced by hand in Arcgis software (ESRI, 2022), and the overlap between *L. fasciola, M. dolomieu*, and each putative model species was assessed using Arcgis software.

### Behavioral analyses

Standardized video recordings of 27 mantle lure-displaying female *L. fasciola* (15 “darter-like” and 12 “leech-like”) were recorded using a Go Pro Hero 6 camera in the summer of 2018 at the two different southeastern Michigan study sites: Sharon Mills County Park (River Raisin) and Hudson Mills Metropark (Huron River). All “darter-like” individuals were grouped together. An additional four video recordings of the lure behavior of sympatric *Lampsilis cardium*, a well studied congener lacking pronounced mantle lure polymorphisms (Kramer, 1970; Haag & Warren, 1999) were collected from the Sharon Mills site to assess interspecific variability in lure behavior. Recordings were captured from a top-down perspective during daylight hours using a standardized frame that included a metric ruler and a Casio TX watch to record date, time, and water temperature data within the video frame. For each displaying female, videos of the lure movements were recorded for 10 min at 120 frames-per-second. Setting up the camera occasionally disturbed the mussels, and video recordings began after waiting some time (usually 2–15 min) until the behavior qualitatively returned to its prior state. Analysis of the videos involved manually recording mantle lure movements for 20,000 frames (2.8 min), starting at 5,000 frames (42 s) to to avoid any camera shaking or hands accidentally blocking the view. The frame numbers when an individual movement began, defined as the first frame where contraction of mantle tissue was observed, and ended, defined as the time that mantle lure returns to it resting state, were noted. Movements of the left and the right mantle lure flaps were recorded seperately.

To quantitatively assess behavioral differences among samples, gait analysis diagrams were created in R for each displaying mussel. Because the lure is mimicking the swimming locomotion of fish, and fish locomotion has been characterized using gait analysis (Liao et al., 2003), we used gait analysis methods to characterize the non-locomotory motions that generate the luring behavior. Averages and standard deviations for the time intervals between lure undulations (the time between the start of one movement and the start of the next) were calculated for each side of each individual, as well as duration undulation (the time between the start of one movement and the end of that movement) and proportion of movements synchronized. Movements were defined as synchronized if the start of a movement on one side was within four frames of the start of a movement on the corresponding side. Proportion of movements synchronized were calculated by dividing the number of synchronized movements by the sum of left movements only, right movements only, and synchronized movements. A Kruskal-Wallis test was used to test for overall differences among lure groups (*L. fasciola* “darter-like”, *L. fasciola “*worm-like”, and *L. cardium*), and pairwise Wilcoxon Signed rank tests were used to compare groups directly with a Bonferroni *p* value adjustment to correct for multiple tests. A Spearman correlation was used to test for an effect of water temperature on time interval between lure undulations.

To further explore differences in lure behavior among groups, we used a general linear mixed model (GLMM), with sample ID as a random factor, to test for differences in lure movement intervals. The GLMM approach, unlike simple mean comparisons, allows the inclusion of all lure movements for all individuals in the model. Because displaying mussels all varied in the number of lure movements recorded over 20,000 frames analyzed, a dataset of 1,000 random bootstrap values was constructed for each individual by randomly sampling values, with replacement. Models were fitted using the ‘lmerTest’ package in R, and Satterthwaite’s (1946) Method (Kuznetsova, Brockhoff & Christensen, 2017) was used to test for significance of fixed effects of lure phenotype on the interval between lure undulations.

## Results

### Captive brood

Two independent classifiers concurred that the 50 preserved specimens from the same maternal brood included 33 “darter-like” (66%) and 17 “worm-like” (34%) individuals (Figs. 3D, 3E and S4).

### ddRAD-seq and phylogenomic analyses

Genomic sequencing returned raw reads ranging from 258,664 to 13,366,692 per individual across the 108 unionid specimens included in the analyses comprising samples of the ingroup *L. fasciola*, sourced from four different populations, along with outgroups *L. cardium* and *Sagittunio nasuta*. Mean coverage depth for the 85% clustering threshold ranged from 2.03 (L_fasciola_AL_mom_2) to 14.25 (L_fasciola_Raisin_16; Table 1). Between 28,725 and 16,161 homologous loci were identified across the two best ddrad datasets (85–50% and 85–75% respectively) and the number of loci recovered was generally consistent among all samples.

**Figure 4:**
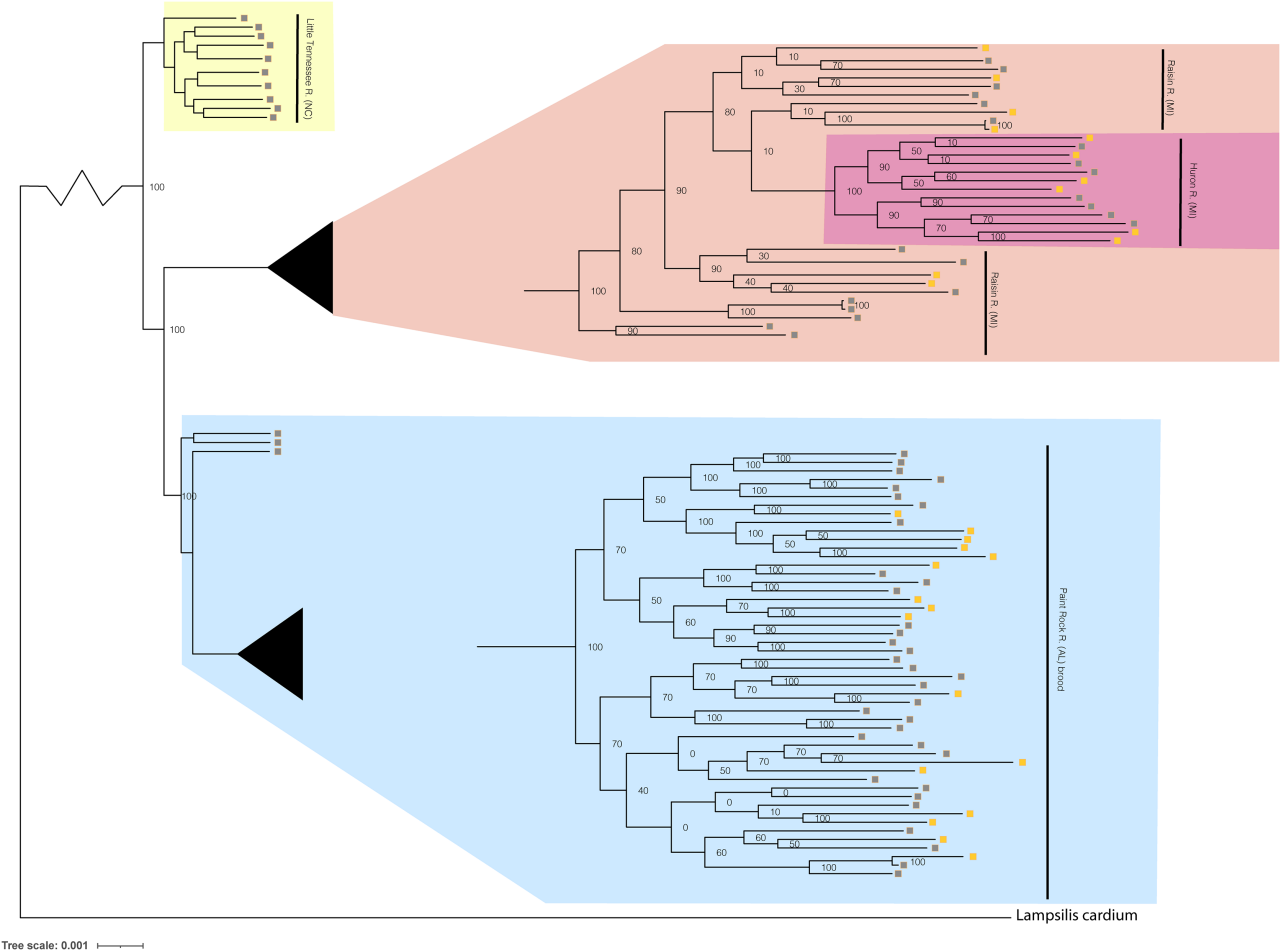
Phylogenomic tree displaying *Lampsilis fasciola* from two MI populations, one NC population, and the Paint Rock River brood raised at the Alabama Aquatic Biodiversity Center. Phylogenomic tree of 96 *Lampsilis fasciola* individuals created in RAxML using 28,735 concatenated ddRAD-seq loci. Gravid, lure-displaying females sampled from two Michigan drainages, River Raisin and Huron River, are respectively highlighted in peach and pink. Specimens sampled from the Paint Rock River, Alabama are highlighted in blue and consisted of three gravid, lure-displaying females, in addition to 50 larval brood members raised at the Alabama Aquatic Biodiversity Center in the zoomed-in tip clade. Gravid, lure-displaying females sampled from the Little Tennessee River in North Carolina are highlighted in yellow. Boxes indicate primary mantle lure phenotypes—“darter-like” (gray) or “worm-like” (orange)—of all *L. fasciola* individuals.

The maximum likelihood tree produced by RAxML (Fig. S4) recovered the following ingroup*/*outgroup topology: (*S. nasuta* (*L. cardium, L. fasciola*)) with outgroup branch lengths greatly exceeding those of the ingroup. To optimize the legibility of ingroup relationships, a compressed, color-coded graphic excluding *S. nasuta* was constructed (Fig. 4). A nested series of phylogenetic relationships was recovered for the four *L. fasciola* fluvial populations with the two Michigan drainages being paraphyletic: (Little Tennessee River (Paint Rock River (River Raisin (River Raisin, Huron River))). The ingroup topology also showed evidence of within-population genealogical relationships with all Paint Rock River brood members forming an exclusive clade (Fig. 4).

The respective primary mantle lure phenotypes—“darter-like” or “worm-like”—of all 92 *L. fasciola* ingroup individuals are indicated in Fig. 4. Note that three of the four population samples—Little Tennessee River, River Raisin and Huron River—were exclusively composed of mantle-lure displaying wild females, and the latter two samples were polymorphic in mantle lure composition. Regarding the Paint Rock River sample, polymorphic lures were restricted to the 50 captive-raised AABC brood members sourced from a gravid, wild female in 2009 (not included in the analyses). The ingroup phylogeny (Fig. 4) contained two polymorphic mantle lure clades, one composed of both Michigan populations (River Raisin and Huron River), the other consisting only of the AABC brood, and both clades had individuals of either lure phenotype interspersed across their respective topologies. Little phylogenetic signal associated with either primary mantle lure phenotype (λ = 0.21; *P* = 0.13).

### Phenotypic ratios over time

**Figure 5:**
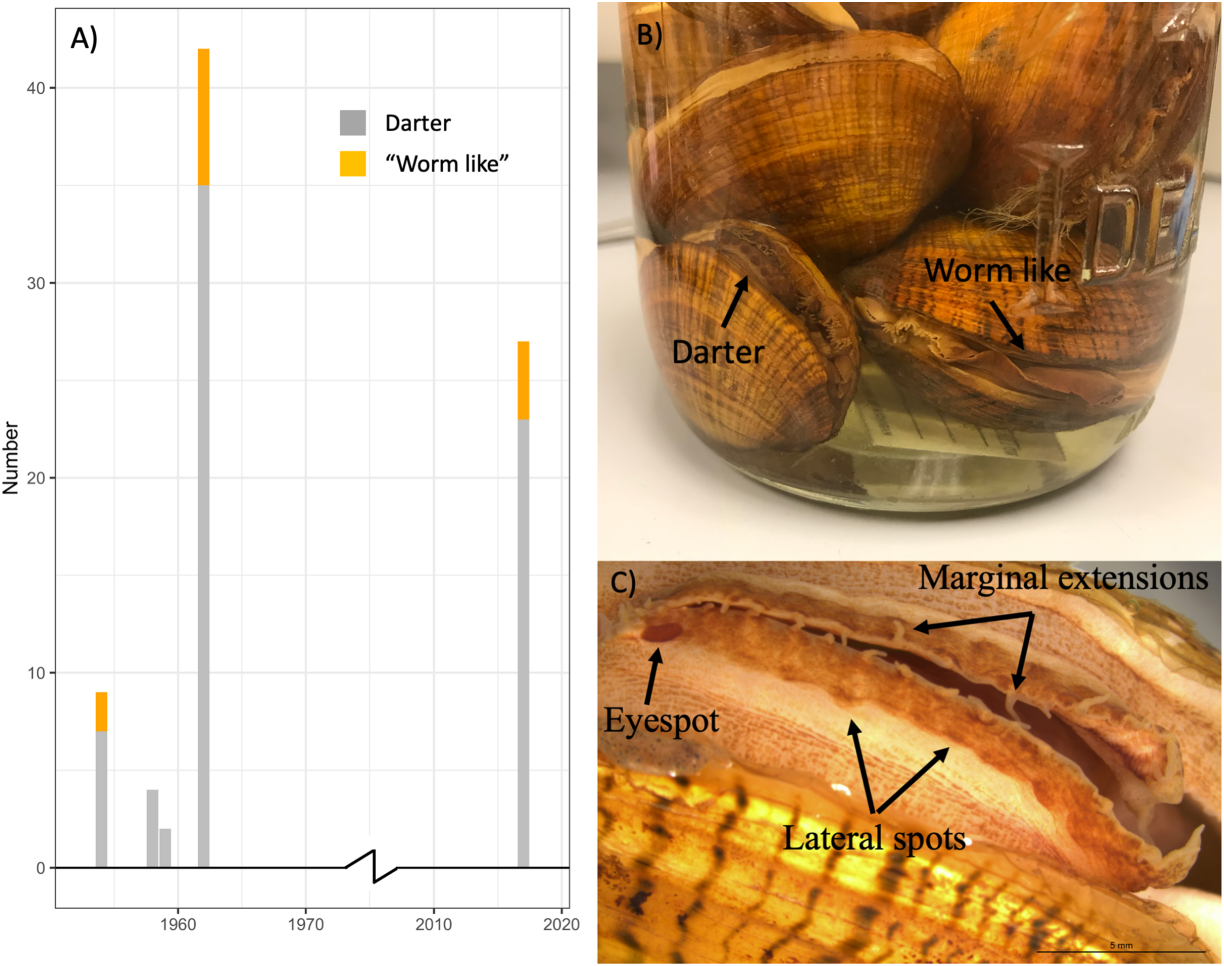
The ratio of “worm-like” and “darter-like” *Lampsilis fasciola* lures over time in the River Raisin, MI, using historical and contemporary samples. The observed frequency of River Raisin *Lampsilis fasciola* primary mantle lure phenotypes (“darter-like”; gray *vs*. “worm-like”; orange) at the Sharon Mills County Park study site during two different time periods. The 1954–1962 data were obtained from the University of Michigan Museum of Zoology (UMMZ) collection specimens, both female and male. The 2017 data were based on field observations of displaying females. (B) A jar of preserved UMMZ Sharon Mills specimens showing a “darter-like” and a “worm-like” mantle lure. (C) A “eyespot”, lateral pigmented blotches, and marginal extensions in a “darter-like” lure of a preserved specimen.

Table S2 summarizes the sex and primary lure phenotypes of 57 *L. fasciola* specimens collected from 1954–1962 at the River Raisin Sharon Mills County Park study site (Fig. 2A) and preserved in the University of Michigan Museum of Zoology’s wet mollusk collection (Figs. 5B and 5C). These historical samples had a collective “darter-like” to “worm-like” ratio of 48:9, with 84.2% of individuals having the more common “darter-like” mantle lure phenotype and 15.8% having the “leech-like” phenotype. Figure 5A contrasts the mid-20^th^ century lure phenotype ratios with a contemporary (2017) estimate in that same population, based on photographic recordings of 27 displaying females. The contemporary ratio was 23:4, with 85.2% of individuals having the more common “darter-like” mantle lure phenotype and 14.8% having the “leech-like” phenotype. The contemporary ratio was not significantly different from the historical ratio (Fisher Exact Test, Χ^2^ = 0.01, *P* = 0.91).

### Putative raisin river lure mimicry models

**Figure 6:**
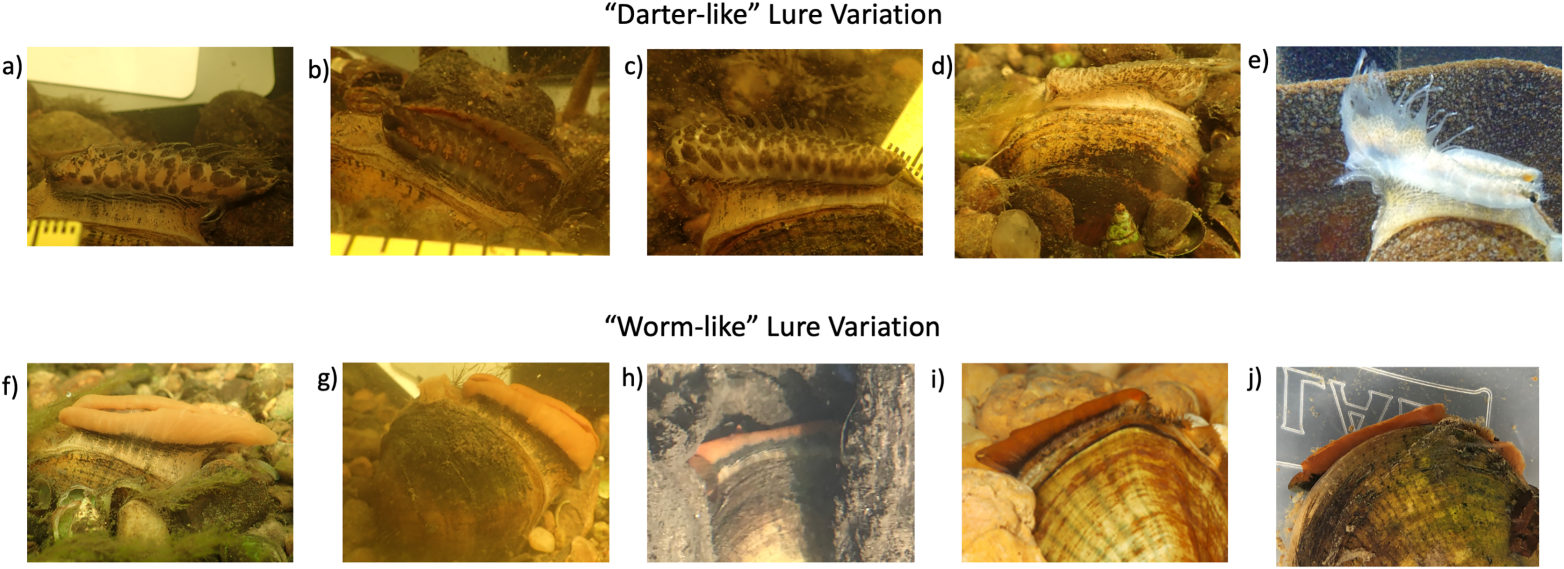
Panel displaying variability in the two primary lure phenotypes of *Lampsilis fasciola*. Variability in lure phenotype, both within a population and across the range of *Lampsilis fasciola*. (A–D) are “darter-like” Raisin River (MI) lures photographed in the field at Sharon Mills County Park. (E) Depicts a “darter-like” lure displayed by a Paint Rock River (AL) female. (F–H) show field photographs of “worm-like” lures displayed by three Sharon Mills females, with specimen H being a younger adult. (I and J) are photographs of two captive AABC specimens, with “worm-like” lures, sourced from the Paint Rock River. The former photo (I), taken in 2011, shows a young (2-year old) female, a member of the captive brood, displaying her lure, and the latter photo (J) is of a female field-sampled in 2022, and showing a partially retracted mantle lure.

The field photographs of 27 displaying female *L. fasciola* mantle lures in the Raisin River Sharon Mills County Park population in 2017 (Fig. S1) were categorized into either “darter-like” (Zanatta, Fraley & Murphy, 2007) or “worm-like” (McNichols, 2007), as summarized in the Materials & Methods section. In addition to the specific features that separate these two primary mantle lure phenotypes (presence/absence of “eyespots” mottled pigmentation, marginal extensions and a “tail”), “darter-like” lures exhibited a much higher degree of variation than did “worm-like” lures, both within populations and across the species range. The latter lure phenotype exhibited a relatively simple, uniform morphology combined with a bright orange coloration underlain with a black basal stripe phenotype in Michigan (Figs. 6F–6H), in Alabama (Figs. 6I and 6J), and in North Carolina populations (Fig. 2A in Zanatta, Fraley & Murphy, 2007). In contrast, Raisin River “darter-like” mantle lures exhibited individual-level variation that was sometime quite marked, especially in details of their pigmentation, and to a more limited degree in their marginal extensions (Figs. 6A–6D and S1). Among individual variation was most pronounced for inter-population camparisons, *e.g*., see the much larger “tail” in the lure displaying Paint Rock River, Alabama specimen shown in Fig. 6E, and also the wider range of phenotypes present in North Carolina populations (Figs. 2B–2D in Zanatta, Fraley & Murphy’s (2007). Male mantle lure rudiment photos are found in Fig. S5.

Despite the considerable individual variation among the 24 photographed Raisin River “darter-like” mantle lures (Fig. S1), it was possible to identify some shared phenotypic motifs, especially in pigmentation pattern, and to informally categorize 23/24 mantle lures with those shared motifs into four general groupings. Group 1 “darter-like” mantle lures were characterized by prominent, chevron-like, darker pigmented blotches, spaced regularily along the flanks of the lure, over a lighter background coloration (Fig. 6A). This general pattern occurred in 7/24 Raisin River “darter-like” lures examined. Group 2 was rarer (3/24 individuals) and consisted of a darker background coloration with large orange blotches spaced regularily along the lure flanks, some divided into “dorsal” and “ventral” elements (Fig. 6B). Group 3 (9/24 individuals) lures were characterized by prominent dark lateral maculation spatially divided into a “ventral” pattern of larger, regularly spaced blotches and a “dorsal” pattern of more numerous, irregular blotches of different sizes (Fig. 6C). Finally, Group 4 (3/24 individuals) lures were characterized by an evenly-dispersed, fine grained freckling of numerous pigmented spots over a lighter background (Fig. 6F).

**Figure 7:**
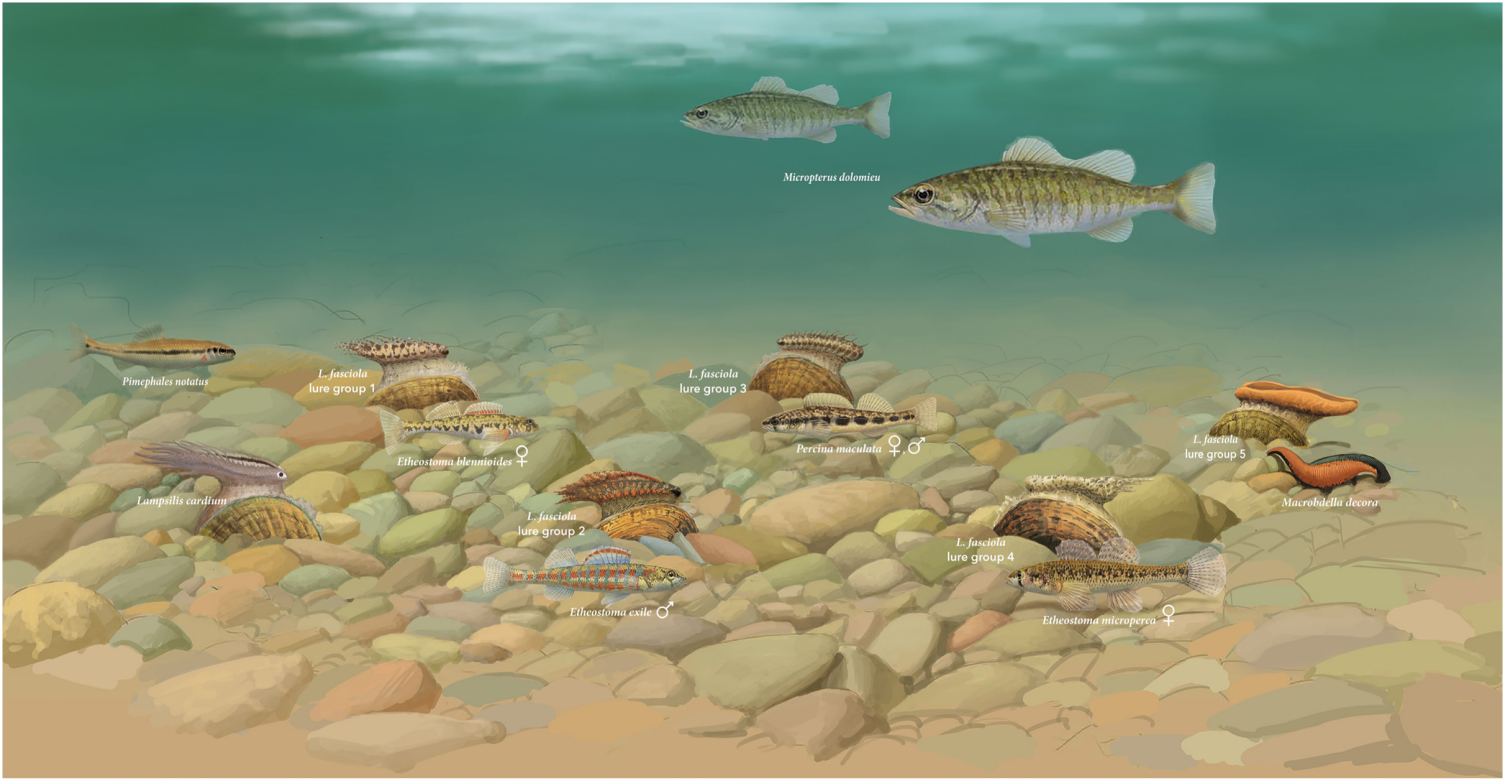
Illustration of hypothetical benthic assemblage of main *Lampsilis fasciola* lure groups, and proposed models. A hypothetical Raisin River (Michigan) benthic assemblage showing displaying exemplars of the putative five main *Lampsilis fasciola* mimetic mantle lure groups (Figs. 6A–6D and 6F) present at the Sharon Mills County Park study site, together with their respective model species, and their primary receiver/fish host, *Micropterus dolomieu*. Also shown is a displaying *Lampsilis cardium* with a “small minnow” mimetic mantle lure (Patterson et al., 2018) and its putative model, *Pimephales notatus*, the most common fish species in the River Raisin (Smith, Taylor & Grimshaw, 1981). Illustration by John Megahan.

To explore putative model species for the four *L. fasciola* Raisin River “darter-like” mantle lure groupings (Figs. 6A–6D and 6F), potential matches (in terms of size, shape and coloration) were sought among the 10 species of Etheosomatidae that occur in the River Raisin (Smith, Taylor & Grimshaw, 1981), many of which display pronounced sexual dimorphism in body coloration (Kuehne & Barbour, 2014). The best apparent matches, depicted in Fig. 7, are as follows: Group 1 (Fig. 6A)-*Etheostoma blennioides* (female coloration*)*, Group 2 (Fig. 6B)-*Etheostoma exile* (male coloration), Group 3 (Fig. 6C)-*Percina maculata* (male and female coloration) and Group 4 (Fig. 6D)-*Etheostoma microperca* (female coloration).

The distinctive color combination of the *L. fasciola* “worm-like” lure-solid orange with a black underlay (Figs. 6F–6J) does not match that of any Raisin River darter, or other Raisin River fishes (Smith, Taylor & Grimshaw, 1981). It does, however, match the coloration and size/shape, of the common North American leech, *Macrobdella decora*, which is widespread and abundant in eastern North America watersheds and typically feeds on aquatic vertebrates (Klemm, 1982; Munro et al., 1992). *M. dolomieu*, *L. fasciola*’s primary host fish, is a generalist predator with a diet of aquatic invertebrates, including leeches, in addition to small fishes (Clady, 1974), and recreational fishers frequently use live and/or artifical leeches as bait to catch this species (Cooke et al., 2022). Based on the available data, it seems that *Macrobdella decora* may be the best model species candidate for the “worm-like” (McNichols, 2007) *L. fasciola* mantle lure phenotype, and will hereafter be referred to as the leech phenotype.

**Figure 8:**
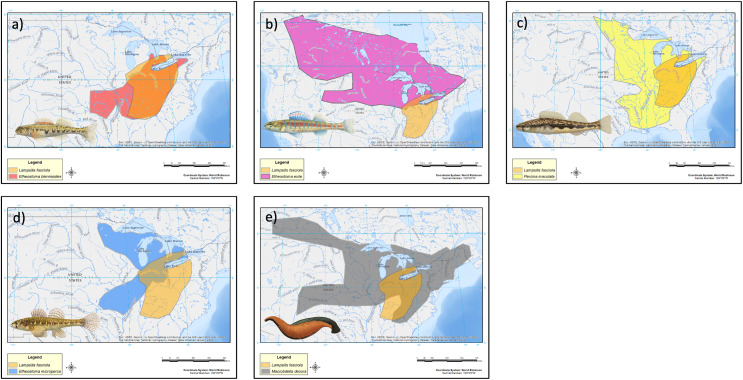
Estimated range maps for proposed models of *Lampsilis fasciola* lures. Estimated range maps for five proposed models for *Lampsilis fasciola* lures compared to the estimated geographic range of *Lampsilis fasciola* (orange). (A) *Etheostoma blennoides* (red), (B) *Etheostoma exile* (mauve), (C) *Percina maculata* (yellow), (D) *Etheostoma microperca* (blue), and (E) *Macrobdella decora* (gray). Note the differences in spatial scales in the panels. Model Illustrations by John Megahan. Base map layers are from U.S. Geological Survey (2022).

The geographic range of the mimic, *L. fasciola*, is a subset of that of its receiver/host *M. dolomieu* (Fig. 2), and the extent of range overlap with all five putative River Raisin mantle lure models were calculated using Arcgis (Table 2) and are shown in Fig. 8. Three of the five putative models-*Etheostoma blenniodes, Percina maculata* and *Macrobdella decorata* have extensive overlap with *L. fasciola*’s range, but *E. exile* and *E. microperca* are restricted to northern portions.

**Table 2:** Estimated range overlap between *Lampsilis fasciola* and five proposed models. The five broad categories of lure phenotypes (Groups a–e) observed at the River Raisin Sharon Mills County Park population of *Lampsilis fasciola* (Fig. 2A), as well as the estimated geographic range overlap between *Lampsilis fasciola* and its five Raisin River putative model species.

Type	Proposed model	Range overlap (km^2^)
Group a	*Etheostoma blennioides*	480,731
Group b	*Etheostoma exile*	87,796
Group c	*Percina maculata*	525,772
Group d	*Etheostoma microperca*	164,539
Group e	*Macrobella decora*	419,259

### Behavioral analyses

**Figure 9:**
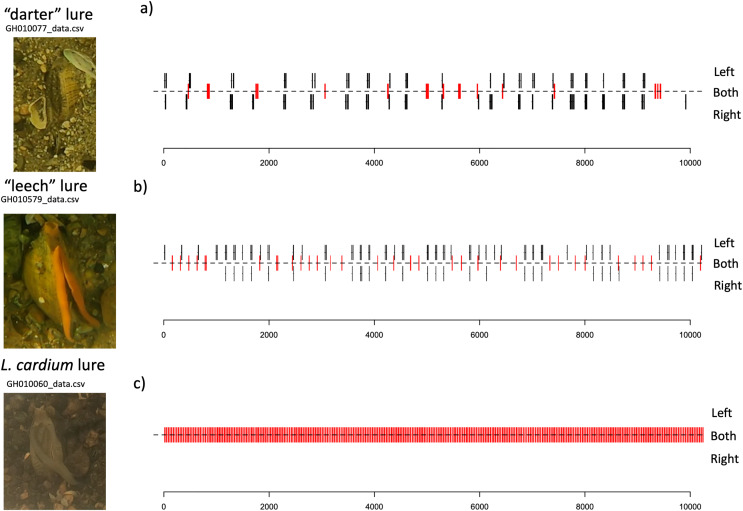
Gait diagrams for three exemplar mussel displays; a “darter-like” *L. fasciola*, a “leech-like” *L. fasciola*, and a *Lampsilis cardium*. Mantle lure gait diagrams for three representative individuals sampled. (A) shows a *Lampsilis fasciola* “darter” lure sample (https://figshare.com/articles/media/GH010077_cropped_mp4Polymorphism_in_the_aggressive_mimicry_lure_of_the_parasitic_freshwater_mussel_Lampsilis_fasciola/24850899), (B) displays a *Lampsilis fasciola* “leech” lure sample (https://figshare.com/articles/media/GH010579_cropped_mp4Polymorphism_in_the_aggressive_mimicry_lure_of_the_parasitic_freshwater_mussel_Lampsilis_fasciola/24850902), and (C) shows a *Lampsilis cardium* sample (https://figshare.com/articles/media/GH010060_cropped_mp4Polymorphism_in_the_aggressive_mimicry_lure_of_the_parasitic_freshwater_mussel_Lampsilis_fasciola/24847932). Red center lines indicate synchronized lure movement for both left and right mantle flaps, and black lines above and below the center line indicate independent left and right movements, respectively. The x-axis denotes time in seconds and frame number (120 fps).

Lure movements for both species consist of small undulations along the length of the mantle lure, beginning about two thirds of the way towards the “tail” side of the lure, and travelling towards the “head” of the lure. The *L. cardium* lure movements always occur on both left and right sides of the mantle lure simultaneosly, while both *L. fasciola* lure phenotyopes exhibit independent movement of the left and right sides of the lure. Qualitatively, *L. fasciola* and *L. cardium* have very different mantle lure display behaviors. Gait diagrams show a clear distinction between *L. cardium* and both primary *L. fasciola* lure phenotypes (“darter” and “leech”). *L. cardium* consistently exhibited a synchronized lure undulation of both mantle lure flaps, whereas *L. fasciola* samples frequently moved left and right mantle flaps independently (Fig. 9 and S6). Gait diagrams also qualitatively showed that *L. fasciola* is charicterized by a high level of variability in undulation interval, *L. cardium* is much more regular in undulation interval with a steady beat frequency.

**Figure 10:**
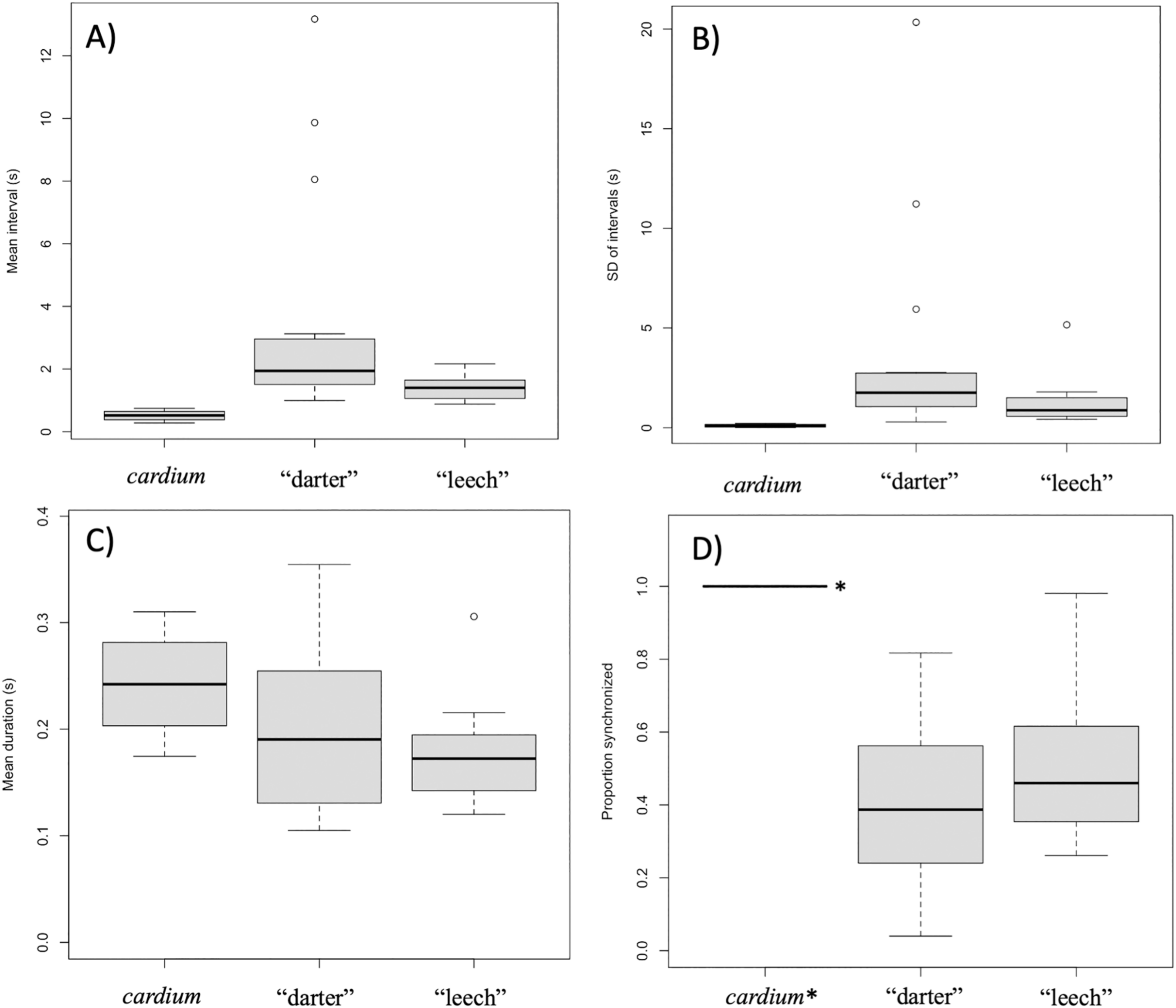
Summary plots for behavioral analysis of the two primary *Lampsilis fasciola* lure phenotypes and *Lampsilis cardium*. Boxplots from behavioral analyses of the two primary *Lampsilis fasciola* mantle lure phenotypes (“darter” *vs*. “leech”, *N* = 15,12 respectively) and of *Lampsilis cardium* (*N* = 4). The middle line in the represents the median, the gray rectangle represents the interquartile range, the whiskers represent the minimum and maximum, excluding outliers, which are defined by 1.5* the interquartile range. (A) Comparison of the mean interval between movements of left mantle flap (s). (B) The standard deviation of lure movement interval (s) as a proxy for variability. (C) The average duration of each mantle lure movement. (D) The proportion of movements that are left-right synchronized. Note that the value for *L. cardium* is 1.0 (all lure movements for all individuals were synchronized) and indicated with *. (A–C) Show means for left mantle flap movements only.

Intervals between movements in *L. cardium* were shorter (Wilcoxon test, W = 0, *N* = 4 *L. cardium*, 15 darter lure *L. fasciola*, 13 leech lure *L. fasciola, p* < 0.01 for both comparisons), less variable (Wilcoxon test, W = 0, *p* < 0.01 for both comparisons) and more synchronized (Wilcoxon test, W = 60, 48, *p* < 0.01 for comparisons with darter and leech lures, respectively) than in *L. fasciola* (Fig. 10). There was no difference in duration of lure undulations between *L. cardium* and both *L. fasciola* lure phenotypes (Wilcoxon test, W = 42,40, *p* = 0.26,0.06 for comparisons with darter and leech lures, respectively). Differences between the lure types of *L. fasciola* were smaller, with inter-movement intervals in the darter phenotype that were longer (Wilcoxon test, W = 142, *p* = 0.01) and marginally non-significantly more variable (W = 128, *p* = 0.07) but similar in duration (Wilcoxon test, W = 97, *p* = 0.76) and degree of synchronization (Wilcoxon test, W = 64, *p* = 0.22, Fig. 10). Table S3 details the time, date, location, temperature and summary statistics of all 34 lure display field recordings.

GLMM were used as an alternative analytical approach that included a large, bootstrapped dataset of lure movements. GLMM results were similar to those of the mean comparisons, with *L. cardium* individuals having shorter movement intervals than either *L. fasciola* lure morphs (an estimated 0.21 s for *L. cardium vs*. 3.2 and 1.0 s, respectively for *L. fasciola* “darter” and “leech” lures). However, these fixed effects are not statistically significant.

## Discussion

Two new pieces of evidence, phylogenomic and genetic, corroborated (Zanatta, Fraley & Murphy’s, 2007) preliminary finding that the primary mantle lure morphs in *L. fasciola* (Figs. 1B and 1C) represent a within-population polymorphism rather than cryptic taxa. In phylogenomic analyses, all three polymorphic population samples (Huron, Raisin, and Paint Rock Rivers), collectively spanning the species range (Figs. 2A–2C), produced tip clades that were comprehensively polyphyletic regarding lure morph type (Fig. 4), and the “darter *vs*. leech” dichotomy yielded a low estimate of phylogenetic signal (λ = 0.21). However, the phylogenomic data did reveal clear evidence of geographic structuring (Fig. 4), with each geographic population forming discrete clades, even among regional populations with a continuous freshwater connection. For example, the Huron and Raisin drainages empty in Western Lake Erie and the Little Tennesse and Paint Rock drainages empty into the Tennessee River (see also VanTassel et al. (2021)). The Paint Rock River (AL) population was sister to the Michigan populations (Fig. 4), a result consistent with phylogeographic associations of multiple other North American species, including unionid mussels and *Micropterus dolomieu*, attributed to hypothesized glacial refugia in the southern Appalachian mountains (Soltis et al., 2006; Borden & Krebs, 2009; Zanatta & Harris, 2013; Hewitt et al., 2018).

Discovery of within-brood mantle lure heterogeneity (Fig. 3), apparently the first such record for unionids, confirms that the *L. fasciola* “darter-like” and “leech-like” mantle lures are polymorphisms rather then cryptic species, corroborating (Zanatta, Fraley & Murphy, 2007), and provides initial, although limited, genetic insights into lure phenotype inheritance. Of the 50 available offspring, the maternal “leech” phenotype was inherited by 17; the remaining 33 had the “darter” phenotype, but none exhibited a recombinant phenotype, *e.g*., “leech” coloration with “darter” marginal extensions or “darter” coloration without marginal extensions. Evidence of discrete, within-brood segregation of the mantle lure polymorphism implies potential control by a single genetic locus and expression of the maternal phenotype in about one third of the offspring is inconsistent with a hypothetical dominant “leech” allele. Additional pedigree insights are currently inhibited by not knowing the number of sires that contributed to the brood: the dam was a wild-mated Paint Rock River individual. Freshwater mussel broods frequently have multiple paternity (Ferguson et al., 2013; Wacker et al., 2018). However, additional analyses of the RADseq dataset are needed to resolve that issue (Thrasher et al., 2018).

There are well-known cases of a single genetic locus controlling a mimic polymorphism in other systems. In butterflies, polymorphic mimicry in wing pigmentation is controlled by an introgressed mimicry supergene in *Heliconius* species (Sheppard et al., 1985; Jay et al., 2018) and by mimicry alleles of the transcription factor *doublesex* (*dsx*) in some *Papilio* species (Palmer & Kronforst, 2020). Note, however, that the *L. fasciola* mantle lure mimicry polymorphism differs in important ways from these butterfly systems. It is more complex because it involves putative models (darters and leeches) from disparate phyla rather than from similar morphospecies (other butterflies), thereby requiring polymorphic trait differentiation in pigmentation and in morphology (Figs. 1B and 1C). It is also a case of aggressive mimicry (Jamie, 2017), different from the Müllerian mimicry of *Heliconius* (Kronforst & Papa, 2015) or the Batesian mimicry of *Papilio* (Kunte, 2009).

Persistence of *L. fasciola* mantle lure polymorphism across a broad geographic scale (Fig. 2) is notable, although the mechanism responsible for widespread maintenace is unclear. One hypothesized mechanism for the persistence of polymorphisms in a species or population is frequency-dependent selection, where fitness is inversly proportional to frequency of a trait (Clarke, 1964; Ayala & Campbell, 1974). Frequency-dependent selection has been observed in other polymorphic mimicry systems (Shine, Brown & Goiran, 2022), and it has been suggested as a possible mechanism for persistence of the *L. fasciola* polymorphism (Zanatta, Fraley & Murphy, 2007; Barnhart, Haag & Roston, 2008; Hewitt, Haponski & Ó Foighil, 2021b). One criterion for frequency-dependent selection is that phenotype ratios oscillate over time as initially rare phenotypes become more successful. However, the historical (1954–1962) and contemporary (2017) data from Sharon Mills County Park (Fig. 5) did not show evidence of such oscillation: the frequencies of the lures (darter lure = 84.2% *vs*. 85.2%, leech lure = 15.8% *vs*. 14.8%) remained essentially the same for both time windows, although we lack data for the intervening years. Theoretically, there are other mechanisms for balancing selection to maintain polymorphisms over long time-scales, including heterozygote advantage or opposing selection pressures favoring different alleles at polymorphic loci (Ford, 1963; Prout, 2000; Mérot et al., 2020), but underlying genetics of the *L. fasciola* polymorphism is unknown at this time, and more data are clearly needed.

The relative uniformity of the “leech” mantle lure phenotype in the River Raisin and throughout the *L. fasciola* range (Figs. 6F–6J) stands in sharp contrast with much higher local and range-wide variation shown by “darter” lures (Figs. 6A–6E). The four putative River Raisin darter model species–*Etheostoma blennioides*, *E. exile*, *E. microperca* and *Percina maculata*–are all common and widespread members of the drainage’s ichthyofauna with 300–900 specimens of each species recovered from 30–100 sampling locations (out of 160 total) by the Smith, Taylor & Grimshaw (1981) ecological survey. That phenotypic lure disparity mirrors the collective phenotypic variability of darters *vs*. *Macrobdella decora*; darters are the second-most diverse fish clade in North America, with ~170 species (Warren & Burr, 1994; Stein & Morse, 2000). Another possibility is that at least some *L. fasciola* “darter-like” lures across the mussel’s range are composite mimics of visual elements from more than one member of their local darter fauna. However, that remains to be established, as does the underlying nature of *L. fasciola* darter lure variation, *i.e*., the degree to which it stems from a continuous spectrum of phenotypes or from the presence of additional discrete polymorphisms. The variability in “darter” lure phenotype does not seem to be associated with any environmental factors, which suggests this variability is not due to ecophenotypic plasticity, although more subtle factors, such as chemical cues, were not measured. Irrespective of the factors promoting variation among *L. fasciola* “darter” lure morphs, maintenance of close phenotypic tracking by lures of their respective models is expected, given host fishes’ strongly adversive reactions to becoming infected (Haag & Warren, 1999).

While the behavior of mantle lures in *L*. mussels has been documented and studied for many decades (Ortmann, 1921; Kramer, 1970; Haag & Warren, 1999), detailed analysis of lure undulation behavior is currently lacking, and the relative importance of behavior *vs*. coloration and morphology is not well understood. The lure undulation for both *L. cardium* and *L. fasciola* starts about two thirds of the way to the “posterior” (“tailed”) side of the lure, and then travels “forward” toward the “eyespot”-bearing “anterior”. This is quite different from the oscilatory “S” shaped anterior-to-posterior swimming movements used by many fishes (Liao, 2007; Smits, 2019). However, it shares some resemblence to the “C” start behavior that many fishes use as an escape mechanism (Witt, Wen & Lauder, 2015). The unusual motion of the mantle lures may therefore be mimicking an escape behavior to some extent, but this remains to be established.

Although the *L. fasciola* behaviors differ significantly from those exhibited by *L. cardium*, there appears to be smaller behavioral polymorphism that distinguish the darter from leech lure phenotypes. Our putative model for River Raisin *L. cardium* mantle lures is a species of pelagic minnow, *Pimephales notatus* (Fig. 7), whose swimming behavior and ecology differs markedly from that of darters (Burress et al., 2017). Darters have lost or greatly reduced their swim bladder and are primarily benthic in habit, spending much of their time resting on the stream bed with slight body movements caused by ambient water flow (Demski, Gerald & Popper, 1973; Zeyl et al., 2016). They intermittently swim by “hopping” across the substrate using pectoral fins and caudal undulations in a manner that is much more erratic than the midwater swimming behavior of most minnows (Winn, 1958; personal observations). This matches a general difference observed between *L. cardium* and *L. fasciola* lures: *L. cardium* lures move faster and more regularly in a highly synchronized way, in contrast with the erratic, often left-right-unsynchronized movements of *L. fasciola* lures, apart from slight passive undulations caused by the ambient river currents. Unfortunately, the sample size of *L. cardium* was low (*N* = 4), despite a great deal of effort, trying to locate gravid female *L. cardium* that were actively displaying.

The only major difference in lure behavior between the “darter” and “leech” lure behvior of *L. fasciola* is a slightly slower rate exhibited by the “darter” lures, and marginally non-significant differences in variability between lure undulations. Both *L. fasciola* morphs have a similar erratic motion, despite the polymorphism putatively modeling taxa from disparate phyla. Leeches swim by a dorsoventeral bending wave moving from head to tail (Jordan, 1998). This swimming behavior is very different from the lure undulations observed in the leech-like *L. fasciola* lures. It is possible that leech behavior differs when moving along the substrate, where displaying *L. fasciola* are located, but we currently lack data on leech swimming behavior in different environments. The ecological importance of the minor, but statistically significant, differences in overall lure beat frequency observed between “darter” and “leech” mimics (Fig. 10) is difficult to evaluate at present, and it remains to be established if it, like the lure morphological differences, is also under genetic control. One additional caveat is that we focused primarily on differences in the timing of mantle lure displays, which were the most practical to measure *in-situ* with the ambient river flow. We also did not have any data on possible chemosensory cues that could potentially be involved.

Our discovery of discrete within-brood inheritance of the *L. fasciola* lure polymorphism is of particular interest because it implies potential control by a single genetic locus. There are a number of parallel cases in the recent literature, *e.g*., in butterflies, the regulation of polymorphic mimicry in wing pigmentation also involves single genetic loci (Jay et al., 2018; Palmer & Kronforst, 2020). Timmermans et al. (2020) used SNP data from *Papilio dardanus* to discover a genomic inversion associated with its mimetic polymorphism, and this approach is likely also tractable for *L. fasciola* given the occurance of polymorphic brood. We are currently raising an additional polymorphic brood at the AABC. Mantle lures are a key adaptive trait in Lampsiline evolution and diversification (Hewitt, Haponski & Ó Foighil, 2021b), and *L. fasciola* is a promising and highly tractable model system to uncover the genetics of lure development and variation in a unionoid mussel.

## Supplemental Information

10.7717/peerj.17359/supp-1 Supplemental Information 1 R code used to create diagrams, manipulate raw behavioral data, and run statistical models.

10.7717/peerj.17359/supp-2 Supplemental Information 2 Average behavioral measurements used in statistical analyses. Summary data on individual mantle lure display field recordings. Video recordings taken during the summer of 2018 at Sharon Mills (Fig. 2a) and Hudson Mills (Fig. 2b). Average movement length and interval were calculated from frame number using a 120fps video recording taken with a Go Pro Hero 6.

10.7717/peerj.17359/supp-3 Supplemental Information 3 Supplementary Figures and Tables.

## Additional Information and Declarations

### Field Study Permissions

The following information was supplied relating to field study approvals (*i.e*., approving body and any reference numbers): Tissue samples from the field were collected under the Michigan Threatened and Endangered Species Collection Permit #TE149.

### DNA Deposition

The following information was supplied regarding the deposition of DNA sequences: The raw demultiplexed ddRAD sequences are available at GenBank: SAMN35800743–SAMN35800847.

### Data Availability

The following information was supplied regarding data availability: The code used to analyze behavioral data, create figures, and run the statistical models is available in the Supplemental File.
