# Supplementary material for: Polymorphism in the aggressive mimicry lure of the parasitic freshwater mussel Lampsilis fasciola
Source: PeerJ. 2024 May 24;12:e17359. doi: 10.7717/peerj.17359 (PMC11129695; doi:10.7717/peerj.17359)
Supplement: Supplemental Information 3 [file peerj-12-17359-s003.pdf]

Supporting Information for

Polymorphism in the aggressive mimicry lure of the parasitic freshwater mussel *Lampsilis fasciola*

Supplementary Table 1: Museum ID numbers, Raw reads, total clusters, and total loci in assembly from the ddRAD sequencing are displayed for each genotyped sample of *Lampsilis fasciola* and of the outgroup taxa. Individual *Lampsilis fasciola* lure phenotype designation followed Zanatta et al. (2007).

Sample Name	Museum ID			Lure Phenotype	Raw reads	Total clusters	Average clustering depth	Loci in assembly
L_fasciola_AL_brood_1	306443	306443-1	306444-1	Worm-like	258664	97681	2.14	483
L_fasciola_AL_brood_2	306444	306443-2	306443-1	Darter-like	5201836	1120710	3.28	25686
L_fasciola_AL_brood_3	306445	306443-3	306444-2	Worm-like	5492519	1126749	3.4	25703
L_fasciola_AL_brood_4	306446	306443-4	306443-2	Darter-like	2429494	632254	2.84	21398
L_fasciola_AL_brood_5	306447	306443-5	306444-3	Worm-like	3152003	760260	3.02	23761
L_fasciola_AL_brood_6	306448	306443-6	306443-3	Darter-like	3212851	810898	2.87	23434
L_fasciola_AL_brood_7	306449	306443-7	306443-4	Darter-like	3649891	593765	4.22	25363
L_fasciola_AL_brood_8	306450	306443-8	306443-5	Darter-like	4869307	1462723	2.29	19089
L_fasciola_AL_brood_9	306451	306443-9	306444-4	Worm-like	3158818	718169	3.08	23033
L_fasciola_AL_brood_10	306452	306443-10	306443-6	Darter-like	4000321	915881	3.12	24916
L_fasciola_AL_brood_11	306453	306443-11	306444-5	Worm-like	5679854	1171842	3.35	25770
L_fasciola_AL_brood_12	306454	306443-12	306443-7	Darter-like	4212783	979265	3.04	24693
L_fasciola_AL_brood_13	306455	306443-13	306444-6	Worm-like	1300563	399134	2.51	12145
L_fasciola_AL_brood_14	306456	306443-14	306443-8	Darter-like	4100372	1043360	2.79	23521
L_fasciola_AL_brood_15	306457	306443-15	306443-9	Darter-like	5804293	1412102	2.91	25570
L_fasciola_AL_brood_16	306458	306443-16	306444-7	Worm-like	1555906	427061	2.7	14099
L_fasciola_AL_brood_17	306459	306443-17	306443-10	Darter-like	2073968	598680	2.59	13668
L_fasciola_AL_brood_18	306460	306443-18	306444-8	Worm-like	6919783	1574429	3.08	25811
L_fasciola_AL_brood_19	306461	306443-19	306443-11	Darter-like	3434210	829507	2.94	23708
L_fasciola_AL_brood_20	306462	306443-20	306443-12	Darter-like	4778853	994416	3.35	25500
L_fasciola_AL_brood_21	306463	306443-21	306444-9	Worm-like	2462560	590095	2.91	20588
L_fasciola_AL_brood_22	306464	306443-22	306444-10	Worm-like	6600876	1406451	3.26	26080
L_fasciola_AL_brood_23	306465	306443-23	306443-13	Darter-like	7090859	1628965	3.06	25932

L_fasciola_AL_brood_24	306466	306443-24	306444-11	Worm-like	4546435	1061394	3	24174
L_fasciola_AL_brood_25	306467	306443-25	306444-12	Worm-like	5379577	1135906	3.35	25703
L_fasciola_AL_brood_26	306468	306443-26	306444-13	Worm-like	5592652	1501130	2.67	23965
L_fasciola_AL_brood_27	306469	306443-27	306444-14	Worm-like	4893957	825855	4.09	25924
L_fasciola_AL_brood_28	306470	306443-28	306443-14	Darter-like	2596873	519103	3.59	22103
L_fasciola_AL_brood_29	306471	306443-29	306443-15	Darter-like	3401334	883485	2.87	21377
L_fasciola_AL_brood_30	306472	306443-30	306444-15	Worm-like	3876395	1014133	2.8	22072
L_fasciola_AL_brood_31	306473	306443-31	306444-16	Worm-like	5391442	1246528	3.07	25009
L_fasciola_AL_brood_32	306474	306443-32	306443-16	Darter-like	4365005	1084596	2.85	23030
L_fasciola_AL_brood_33	306475	306443-33	306443-17	Darter-like	5116507	1117916	3.16	24667
L_fasciola_AL_brood_34	306476	306443-34	306443-18	Darter-like	7480755	1601100	3.19	26163
L_fasciola_AL_brood_35	306477	306443-35	306443-19	Darter-like	8121426	1825135	3.02	25972
L_fasciola_AL_brood_36	306478	306443-36	306443-20	Darter-like	5521997	1414238	2.78	24163
L_fasciola_AL_brood_37	306479	306443-37	306443-21	Darter-like	6562641	1579514	2.88	25476
L_fasciola_AL_brood_38	306480	306443-38	306443-22	Darter-like	6303766	1596624	2.76	24448
L_fasciola_AL_brood_39	306481	306443-39	306443-23	Darter-like	6206795	1488925	2.91	24648
L_fasciola_AL_brood_40	306482	306443-40	306443-24	Darter-like	8630897	1891164	3.11	26176
L_fasciola_AL_brood_41	306483	306443-41	306443-25	Darter-like	7293683	1716571	2.95	25604
L_fasciola_AL_brood_42	306484	306443-42	306443-26	Darter-like	4896252	1193262	2.88	22829
L_fasciola_AL_brood_43	306485	306443-43	306443-27	Darter-like	6098052	1471714	2.9	25074
L_fasciola_AL_brood_44	306486	306443-44	306443-28	Darter-like	7495994	1698871	3.04	25701
L_fasciola_AL_brood_45	306487	306443-45	306443-29	Darter-like	3937758	670698	4.06	24947
L_fasciola_AL_brood_46	306488	306443-46	306443-30	Darter-like	6370942	1343655	3.26	25855
L_fasciola_AL_brood_47	306489	306443-47	306443-31	Darter-like	5542864	1318463	2.96	24550
L_fasciola_AL_brood_48	306490	306443-48	306443-32	Darter-like	6313913	1469606	2.98	24983
L_fasciola_AL_brood_49	306491	306443-49	306443-33	Darter-like	3163000	789239	2.9	24776
L_fasciola_AL_brood_50	306492	306443-50	306443-34	Darter-like	1728370	548529	2.35	17837
L_fasciola_Huron_5	306493	306444-1	306445-1	Darter-like	953302	259898	2.8	10996
L_fasciola_Huron_6	306494	306444-2	306446-1	Worm-like	1682931	362706	3.31	16809

L_fasciola_Huron_7	306495	306444-3	306446-2	Worm-like	746944	157212	3.29	10644
L_fasciola_Huron_8	306496	306444-4	306446-3	Worm-like	1899689	402515	3.25	16584
L_fasciola_Huron_9	306497	306444-5	306445-2	Darter-like	1213655	293090	2.97	11818
L_fasciola_Huron_10	306498	306444-6	306445-3	Darter-like	7775910	1275602	3.87	22035
L_fasciola_Huron_11	306499	306444-7	306445-4	Darter-like	1533281	295767	3.55	15386
L_fasciola_NC_1	306500	306445-1	306447-1	Darter-like	1308813	254002	3.61	11873
L_fasciola_NC_2	306501	306445-2	306447-2	Darter-like	4862573	852380	3.77	18321
L_fasciola_NC_3	306502	306445-3	306447-3	Darter-like	663874	165869	2.95	9960
L_fasciola_NC_4	306503	306445-4	306447-4	Darter-like	2610453	465228	3.76	13790
L_fasciola_NC_5	306504	306445-5	306447-5	Darter-like	6927947	1459334	3.05	20804
L_fasciola_NC_6	306505	306445-6	306447-6	Darter-like	1051195	202171	3.27	12415
L_fasciola_NC_7	306506	306445-7	306447-7	Darter-like	1948092	382878	3.61	17101
L_fasciola_NC_8	306507	306445-8	306447-8	Darter-like	3475751	669278	3.69	20683
L_fasciola_NC_9	306508	306445-9	306447-9	Darter-like	5693936	1634946	2.46	22325
L_fasciola_NC_10	306509	306445-10	306447-10	Darter-like	2175381	464794	3.38	17094
L_fasciola_NC_11	306510	306445-11	306447-11	Darter-like	2189933	516643	3.05	17580
L_fasciola_Redo_1	306511	306446-1	306448-1	Darter-like	1455864	327622	2.62	13478
L_fasciola_Redo_2	306512	306446-2	306448-2	Darter-like	1839020	436418	2.43	13181
L_fasciola_Raisin_2	306513	306447-1	306449-1	Darter-like	8235827	1716137	3.29	25555
L_fasciola_Raisin_3	306514	306447-2	306449-2	Darter-like	6032935	1488448	2.85	25006
L_fasciola_Raisin_4	306515	306447-3	306449-3	Darter-like	12947164	3587458	2.45	25245
L_fasciola_Raisin_1	306516	306447-4	306449-4	Darter-like	6639384	1086218	3.97	23458
L_fasciola_Raisin_5	306517	306447-5	306449-5	Darter-like	10059843	1997619	3.41	25363
L_fasciola_Raisin_6	306518	306447-6	306449-6	Darter-like	8019689	1847955	3.01	25769
L_fasciola_Raisin_7	306519	306447-7	306449-7	Darter-like	3816242	681697	3.95	24606
L_fasciola_Raisin_8	306520	306447-8	306449-8	Darter-like	6117037	1282299	3.27	22439
L_fasciola_Raisin_9	306521	306447-9	306450-1	Worm-like	5170380	775979	4.64	25798
L_fasciola_Raisin_10	306522	306447-10	306449-9	Darter-like	761451	176858	3.14	11477
L_fasciola_Raisin_11	306523	306447-11	306450-2	Worm-like	7140657	1670143	2.97	25519

L_fasciola_Raisin_12	306524	306447-12	306449-10	Darter-like	890521	203114	2.91	10582
L_fasciola_Raisin_13	306525	306447-13	306449-11	Darter-like	1071361	225030	3.47	13512
L_fasciola_Raisin_14	306526	306447-14	306449-12	Darter-like	3644379	946273	2.82	21995
L_fasciola_Raisin_15	306527	306447-15	306449-13	Darter-like	3578043	482446	5.04	17514
L_fasciola_Raisin_16	306528	306447-16	306449-14	Darter-like	2351544	114072	14.25	516
L_fasciola_Raisin_17	306529	306447-17	306449-15	Darter-like	5272816	1304726	2.87	23305
L_fasciola_Huron_1	306530	306448-1	306452	Worm-like	13366692	4050829	2.26	17555
L_fasciola_Huron_2	306531	306448-2	306451-1	Darter-like	2819896	928226	2.24	20205
L_fasciola_Huron_3	306532	306448-3	306451-2	Darter-like	662275	186602	2.66	7653
L_fasciola_Huron_4	306533	306448-4	306451-3	Darter-like	4792093	855457	3.88	24512
L_fasciola_AL_mom_1_10	306534	306449-1	306453-1	Darter-like	8095030	1840917	2.95	25420
L_fasciola_AL_mom_2_21	306535	306449-2	306453-2	Darter-like	10329331	3504027	2.03	24488
L_fasciola_AL_mom_3_16	306536	306449-3	306453-3	Darter-like	10384477	2987559	2.34	25056
L_fasciola_Huron_12	306537	306450-1	306454-1	Worm-like	6906349	1672394	2.87	25281
L_fasciola_Huron_13	306538	306450-2	306454-2	Worm-like	6955496	1670627	2.88	25593
L_fasciola_Raisin_18	306539	306450-3	306454-3	Worm-like	5506215	1301878	3	25373
L_fasciola_Raisin_19	306540	306451-1	306455-1	Worm-like	6611596	1524682	3.03	25604
L_fasciola_Raisin_20	306541	306451-2	306455-2	Worm-like	4894495	1276608	2.74	24931
L_fasciola_Raisin_21	306542	306451-3	306455-3	Worm-like	8396562	1736736	3.26	25490
L_cardium_1	306543	306452-1	306456-1		6864226	1710220	2.8	14625
L_cardium_2	306544	306452-2	306456-2		4898330	1091622	3.11	13433
L_cardium_3	306545	306452-3	306456-3		7109883	2005565	2.5	14563
L_cardium_4	306546	306452-4	306456-4		4637077	997208	3.27	13860
S_nasuta_1	306547	306453	306457		4544989	1169260	2.55	10441

Supplementary Table 2: Summary of the sex and lure phenotypes of all 57 University of Michigan Museum of Zoology *Lampsilis fasciola* individuals present in 8 separate mid-20th century collections made from the River Raisin at Sharon Mills County Park (Fig. 2a).

Collection Date	Male darter-like	Male worm-like	Female darter-like	Female worm-like	Total darter-like	Total worm-like	Total
9/30/54	2	1	5	1	7	2	9
5/21/58	0	0	4	0	4	0	4
7/28/59	0	0	2	0	2	0	2
4/24/62	3	0	3	0	6	0	6
5/10/62	5	1	4	1	9	2	11
6/19/62	4	2	1	0	5	2	7
6/25/62	3	1	2	0	5	1	6
7/20/62	5	1	5	1	10	2	12

Supplementary Table 3: Summary data on individual mantle lure display field recordings. Video recordings were taken during the summer of 2018 at Sharon Mills (Fig. 2a) and Hudson Mills (Fig. 2b). Average movement length and interval was calculated from frame number from a 120fps video recording using a Go Pro Hero 6.

File	Average Movement duration (L)	Average Movement duration (R)	Average Interval (L)	Average Interval (R)	Interval Standard Deviation (L)	Interval Standard Deviation (R)	Proportion of Movements Synchronized	Lure Phenotyp e	Time	Temperature °C	Date	Site
GH010073	0.18	0.18	1.50	1.99	1.28	1.65	0.44	"leech"	11:26	16.4	7/10/18	Hudson Mills
GH010074	0.14	0.14	1.72	1.69	1.73	1.73	0.56	"leech"	14:09	18.5	7/9/18	Sharon Mills
GH010599	0.22	0.23	13.17	8.71	20.34	19.34	0.04	"darter"	11:29	18.3	7/10/18	Hudson Mills
GH010601	0.21	0.22	1.02	1.25	0.52	0.33	0.48	"leech"	NA	NA	7/10/18	Hudson Mills
GH010602	0.17	0.17	1.19	1.52	0.62	0.69	0.32	"leech"	13:36	20.6	7/10/18	Hudson Mills
GH010603	0.23	0.21	1.20	1.14	1.22	1.10	0.24	"darter"	13:57	20.5	7/10/18	Hudson Mills
GH010075	0.15	0.15	1.77	1.58	1.79	1.78	0.27	"leech"	2:48	18.2	7/9/18	Sharon Mills
GH010598	0.27	0.27	1.80	1.75	1.19	1.17	0.80	"darter"	11:18	18.2	7/10/18	Hudson Mills
GH010597	0.31	0.30	1.30	1.27	0.57	0.50	0.93	"leech"	11:06	18.1	7/10/18	Hudson Mills
GH010595	0.22	0.22	1.51	1.99	1.17	1.04	0.42	"leech"	10:34	17.6	7/10/18	Hudson Mills
GH010056	0.24	0.24	3.12	3.00	1.89	2.26	0.38	"darter"	12:59	21.3	6/11/18	Sharon Mills
GH010077	0.19	0.22	1.83	1.66	1.91	1.76	0.18	"darter"	11:26	16.4	7/10/18	Hudson Mills
GH010055	0.29	0.29	2.34	2.34	1.32	1.31	0.60	"darter"	11:59	21.8	6/11/18	Sharon Mills
GH010016	0.35	0.37	1.83	1.81	0.50	0.47	0.53	"darter"	NA	NA	6/12/18	Sharon Mills
GH010057	0.30	0.26	9.87	7.40	11.22	8.65	0.30	"darter"	13:16	21.4	6/11/18	Sharon Mills
GH010593	0.15	0.15	0.99	0.88	0.58	0.59	0.39	"leech"	14:09	20.7	7/9/18	Sharon Mills
GH010062	0.14	0.14	2.27	2.77	1.76	1.84	0.24	"darter"	10:21	19.2	7/4/18	Hudson Mills
GH010064	0.17	0.18	1.10	1.10	0.42	0.42	0.98	"leech"	NA	20.8	7/4/18	Hudson Mills
GH010065	0.12	0.13	1.21	1.66	0.92	0.83	0.45	"darter"	14:23	21	7/4/18	Hudson Mills
GH010063	0.14	0.14	1.05	1.05	0.29	0.31	0.82	"darter"	11:13	19.6	7/4/18	Hudson Mills
GH010579	0.12	0.12	0.88	1.29	0.55	0.47	0.26	"leech"	11:02	20	7/4/18	Hudson Mills
GH010580	0.13	0.13	1.56	1.82	1.13	1.43	0.67	"leech"	11:52	21.3	7/4/18	Hudson Mills
GH010581	0.18	0.18	2.17	2.51	5.16	5.67	0.54	"leech"	13:36	23.3	7/4/18	Hudson Mills
GH010582	0.10	0.11	1.94	2.16	2.76	2.79	0.19	"darter"	14:18	23.4	7/4/18	Hudson Mills
GH010583	0.11	0.12	0.99	1.12	0.91	0.82	0.60	"darter"	14:52	NA	7/4/18	Hudson Mills
GH010068	0.12	0.11	2.79	2.73	2.71	2.37	0.41	"darter"	NA	NA	6/12/18	Sharon Mills
GH010048	0.15	0.14	8.05	7.67	5.94	5.60	0.39	"darter"	NA	NA	6/7/18	Sharon Mills
GH010060	0.17	0.17	0.28	0.28	0.01	0.01	1.00	cardium	NA	NA	6/5/20	Sharon Mills
GH010163	0.23	0.23	0.55	0.55	0.12	0.12	1.00	cardium	NA	NA	6/5/20	Sharon Mills
GH010620	0.25	0.25	0.48	0.48	0.05	0.05	1.00	cardium	NA	NA	5/31/21	Sharon Mills
GH010618	0.25	0.25	0.41	0.41	0.10	0.10	1.00	cardium	NA	NA	6/1/21	Sharon Mills

Group 1

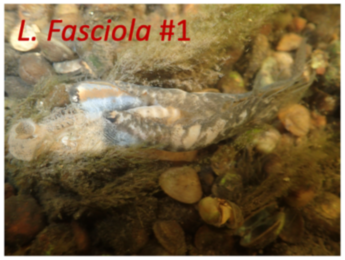

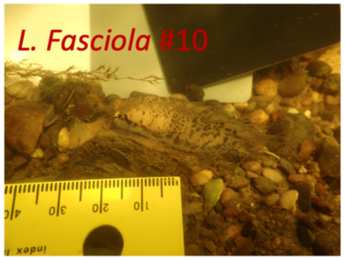

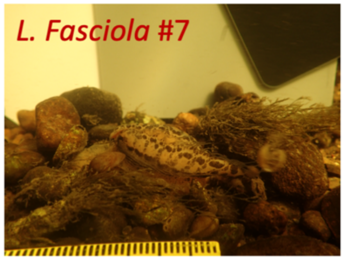

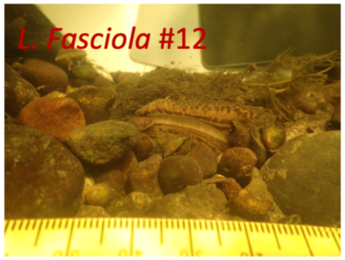

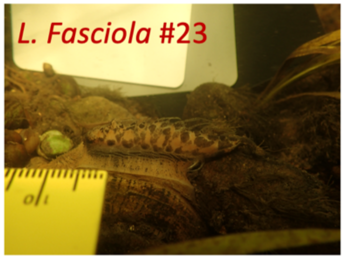

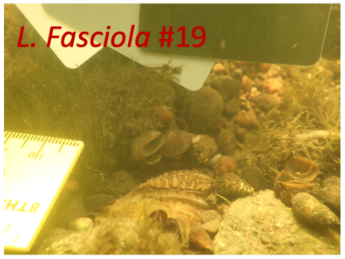

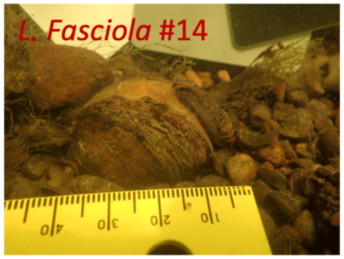

Group 2

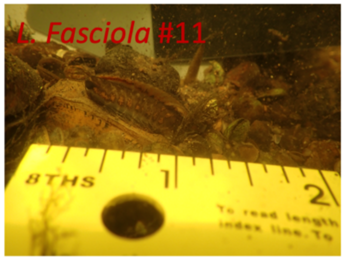

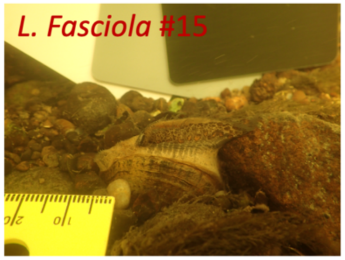

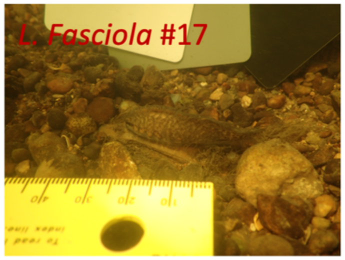

Group 3

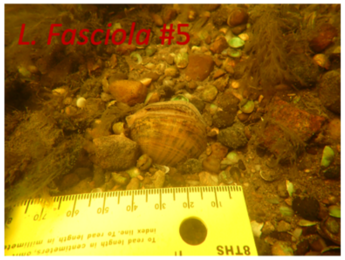
L. Fasciola #5

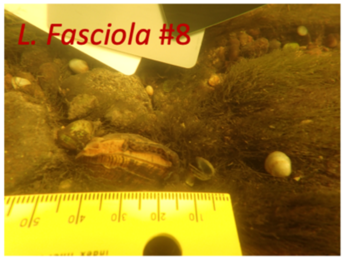
L. Fasciola #8

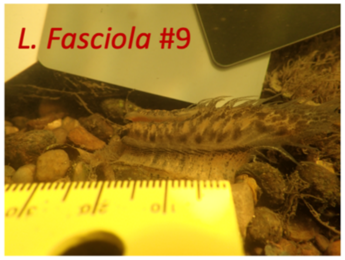
L. Fasciola #9

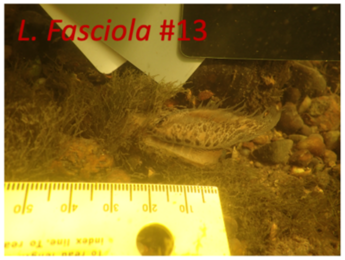
L. Fasciola #13

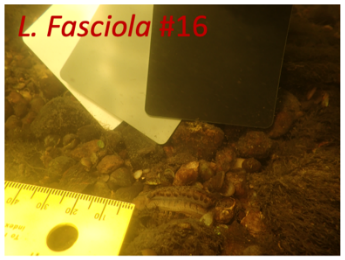
L. Fasciola #16

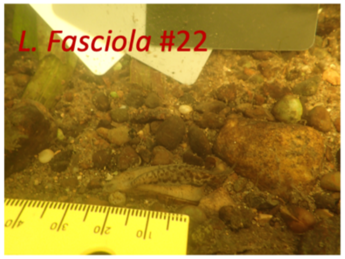
L. Fasciola #22

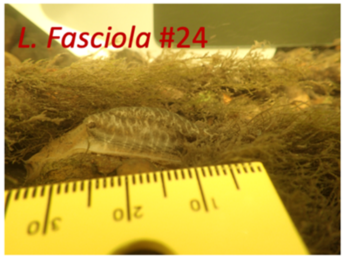
L. Fasciola #24

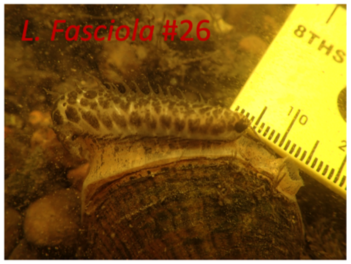
L. Fasciola #26
8THS

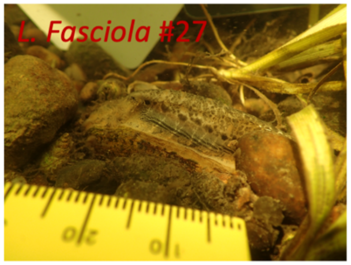
L. Fasciola #27

Group 4

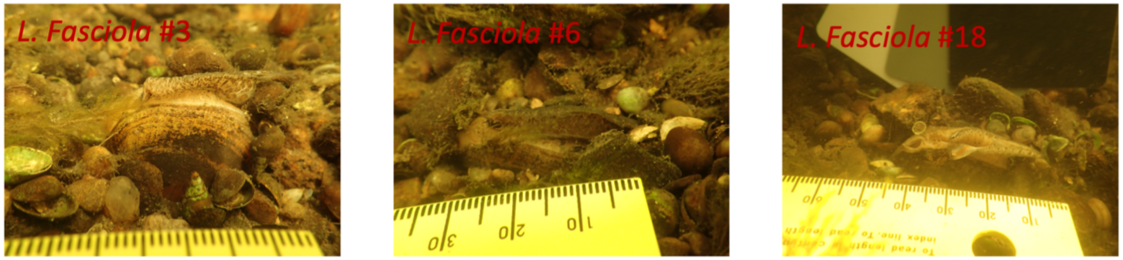

Group 5

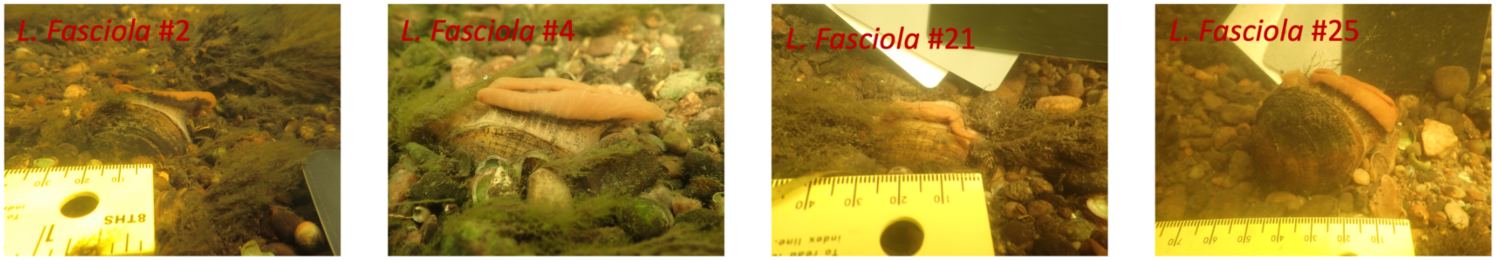

Supplementary Figure 1: Photographs from 27 *Lampsilis fasciola* lures taken at Sharon Mills (Fig. 2a) in Summer of 2017. Groups are defined by morphological similarity and individual numbers refer to order in which photographs were taken.

Mussel brood notes

Date : 7-13-09

Species: L. fasciola

Source: Paint Rock R.

ID number	Length (mm)	Width (mm)	Height (mm)	Mass (g)
LF01	54	26	38	32.32

* Bright orange and black mantle lure *

A. Suspension volume (ml): 250 (Adjust to give ~20 per drop)

Drop #	Undeveloped eggs	Open glochidia	Closed glochidia	Open after salt
1		29	Ø	1
2		28	1	1
3		22	1	1
4		20	1	1
5		23	3	Ø
6		17	1	1
7		18	Ø	Ø
8		29	3	Ø
9		26	2	Ø
10		26	4	1
**Sums**	B	C 238	D 16	E 6

Total glochidia = (C + D) * A/2 = 31750

Viable glochidia = (C - E) * A/2 = 29000 — 91% viable glo.

7.25 L

29 REB

Supplementary Figure 2: Alabama Aquatic Biodiversity Center data sheet documenting the mantle lure phenotype (bracketed with red *s) and the larval brood size of the gravid Paint Rock River female *Lampsilis fasciola* used to establish the inaugural AABC cultured unionid brood in 2009.

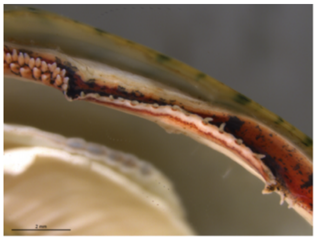

Darter_01

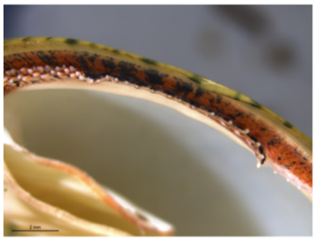

Darter_02

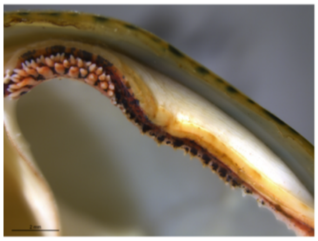

Darter_03

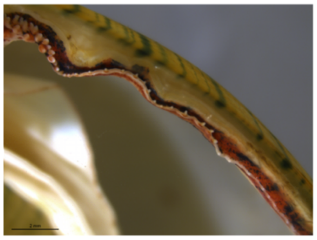

Darter_04

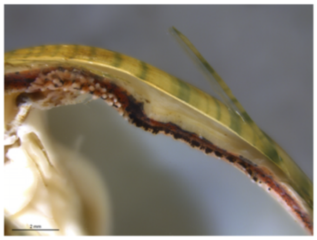

Darter_05

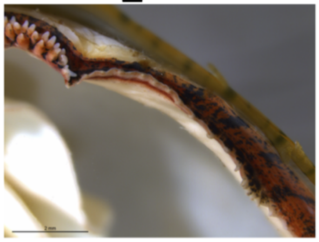

Darter_06

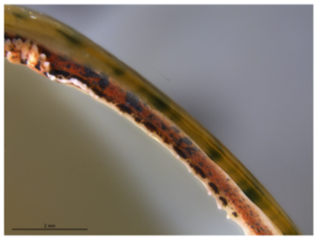

Darter_07

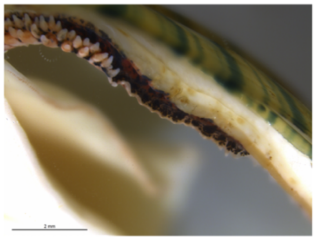

Darter_08

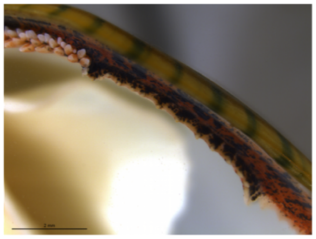

Darter_09

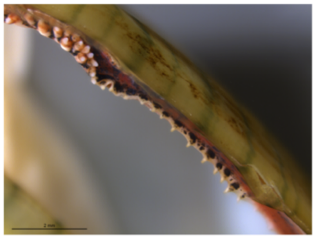

Darter_10

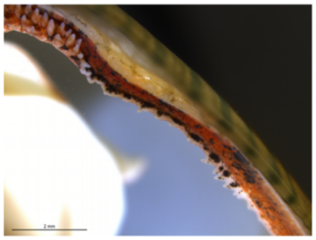

Darter_11

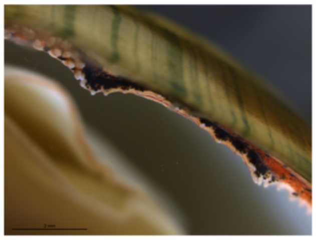

Darter_12

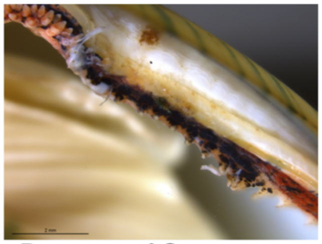

Darter_13

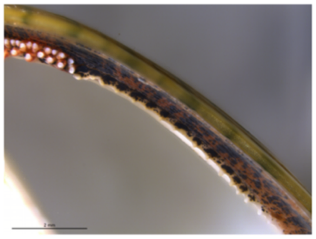

Darter_14

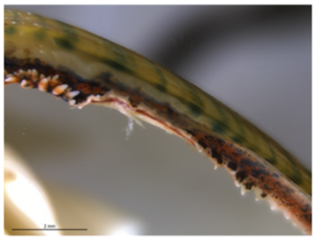

Darter_15

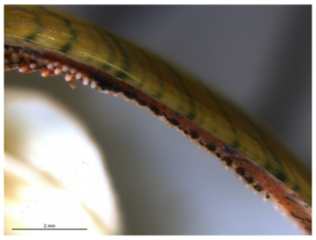

Darter_16

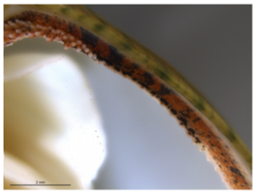
Darter_17

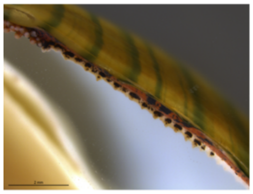
Darter_18

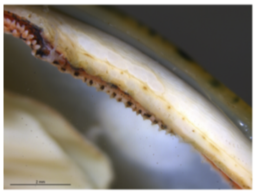
Darter_19

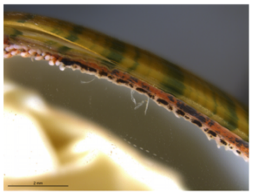
Darter_20

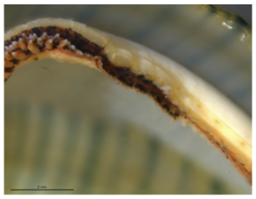
Darter_21

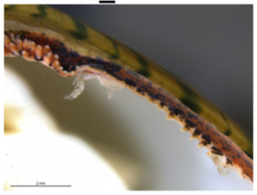
Darter_22

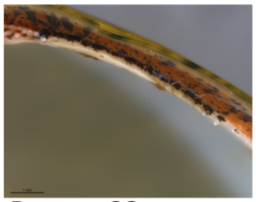
Darter_23

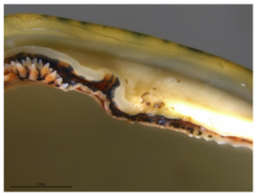
Darter_24

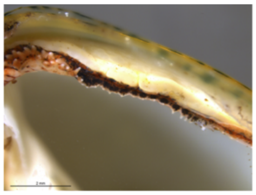
Darter_25

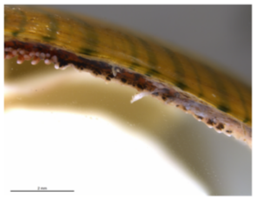
Darter_26

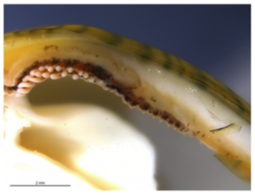
Darter_27

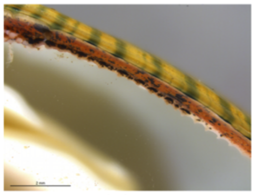
Darter_28

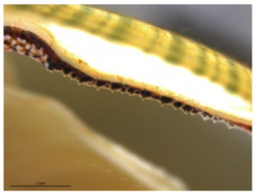
Darter_29

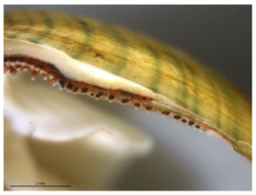
Darter_30

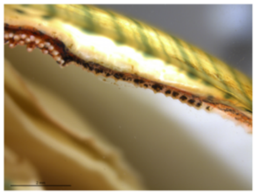
Darter_31

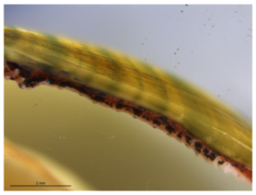
Darter_32

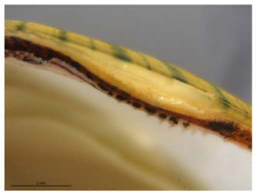
Darter_33

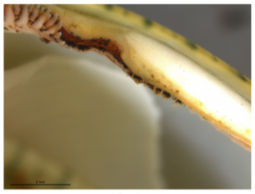
Darter_34

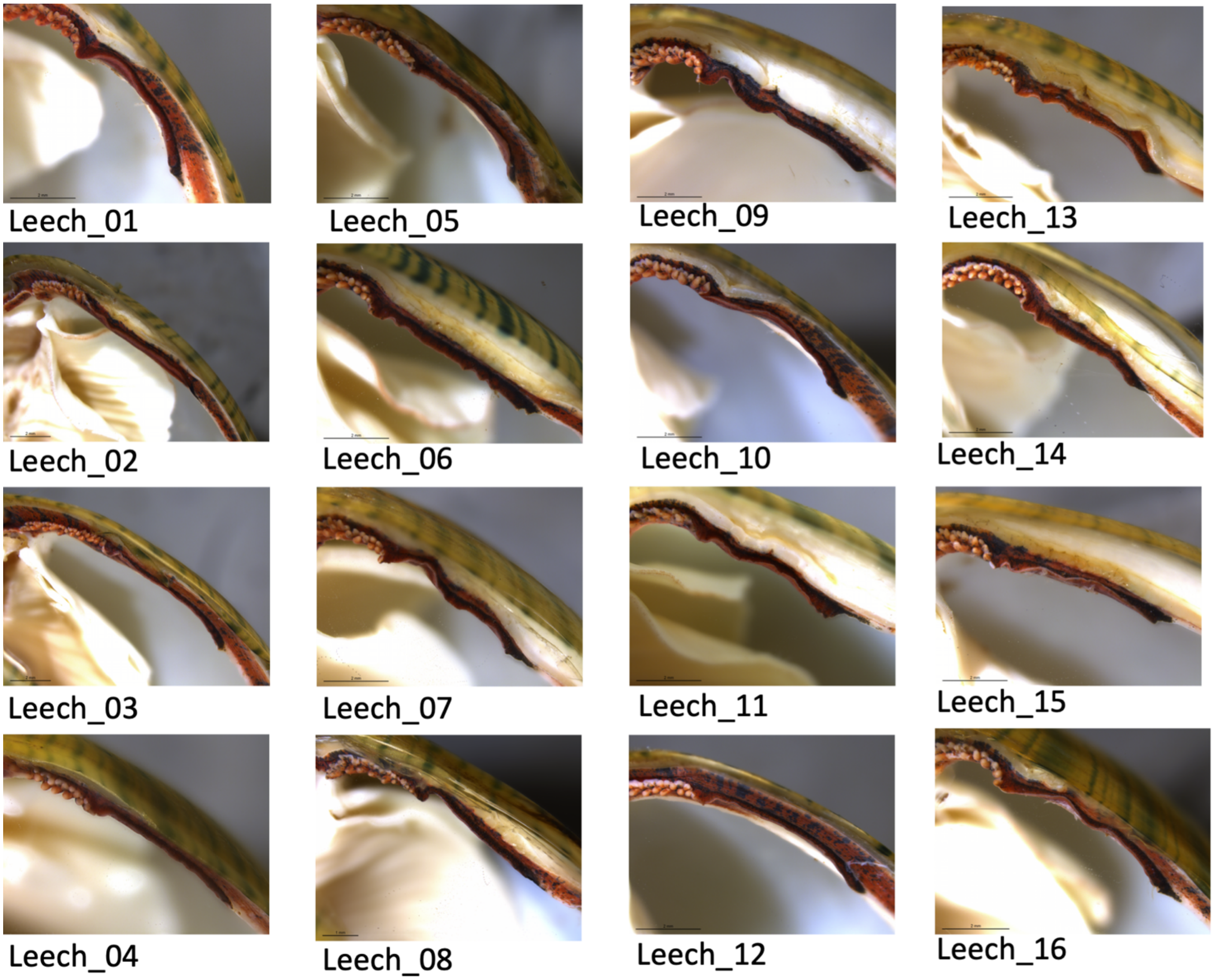

Supplementary Figure 3: Photographs of *Lampsilis fasciola* lure structure taken from 50 full or half-siblings raised from a single gravid female at the Alabama Aquatic Biodiversity Center in 2009. Each individual is categorized based on whether it has a darter-like or worm-like lure phenotype.

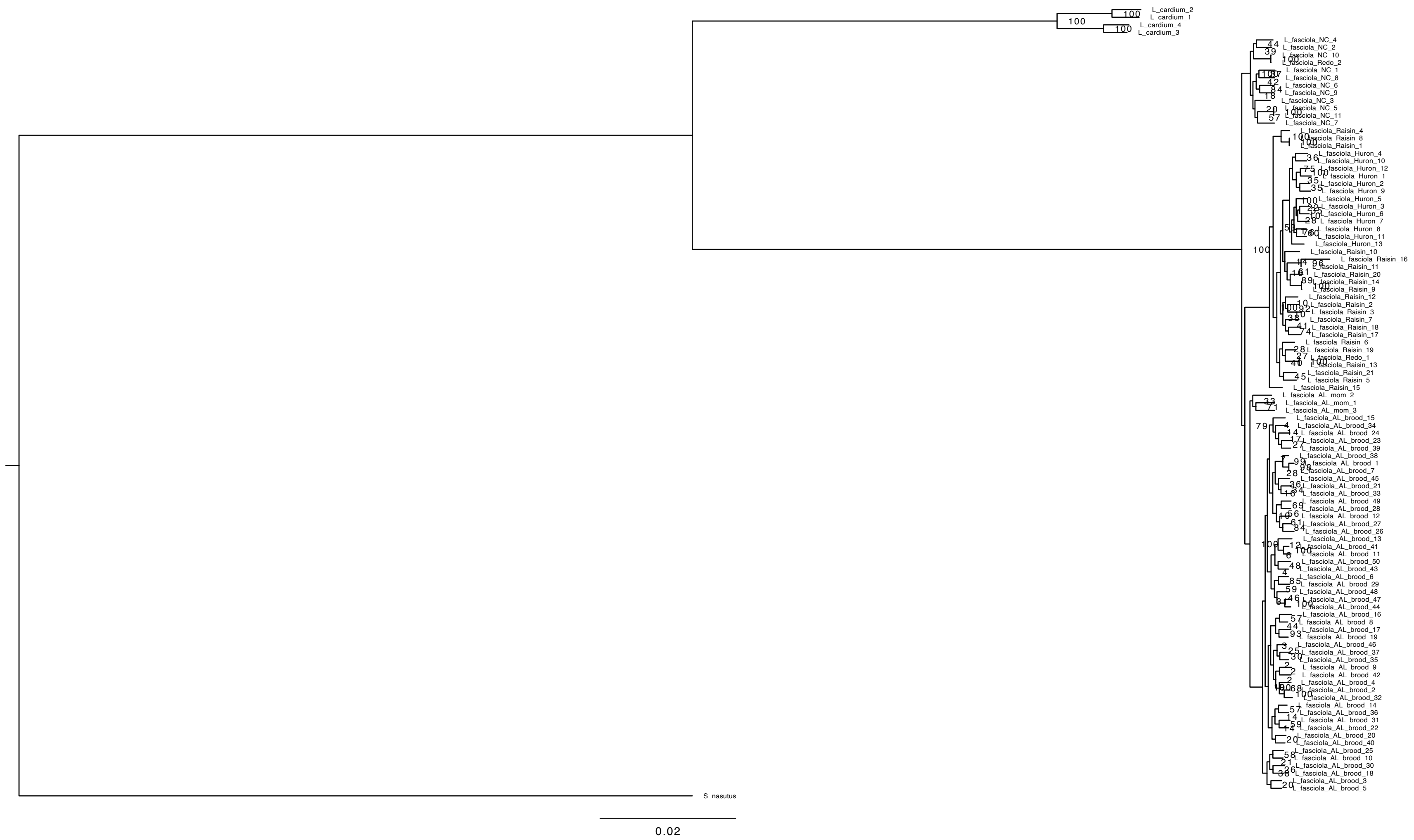

Supplementary Figure 4: Maximum likelihood phylogeny of *Lampsilis fasciola* mussels created with RAxML v8.2.8 using a general time reversible model. Support for each node was determined using 100 fast parametric bootstrap replications. Bootstrap values are adjacent to each node. Scale bar represents mean number of base pair substitutions per site.

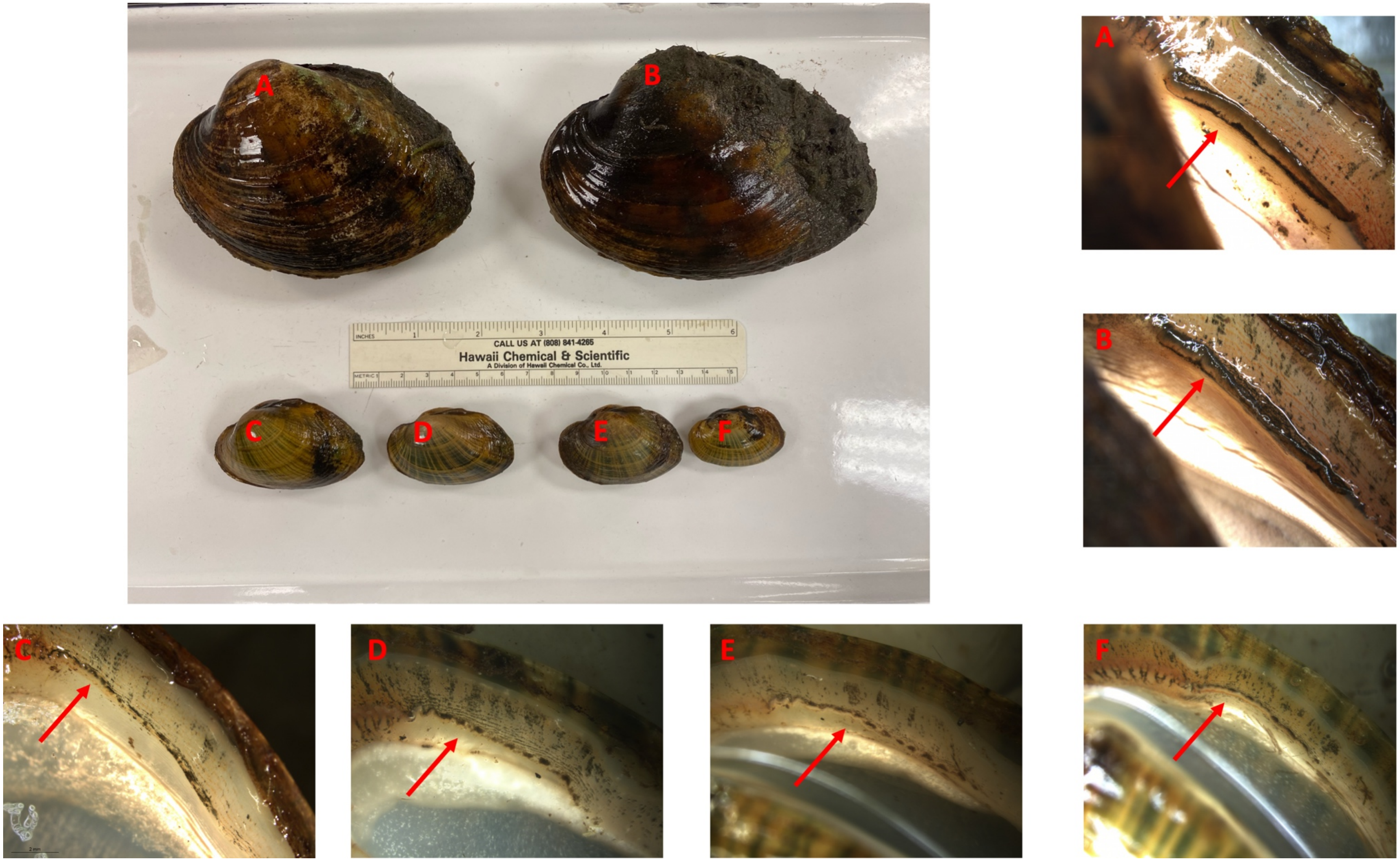

Supplementary Figure 5: A group photograph of 6 River Raisin Sharon Mills (MI) male lampsiline mussels - 2 *Lampsilis cardium* (A, B) and 4 *Lampsilis fasciola* (C-F) taken in May 2023 - together with individual photographs of their respective right mantle lure rudiments (arrows). All 4 *L. fasciola* mantle lures were "darter-like" with mottled coloration and minute marginal extensions.

GH010599

Darter

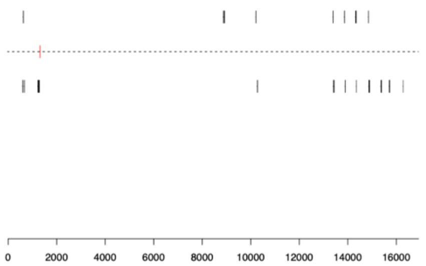

GH010603

Darter

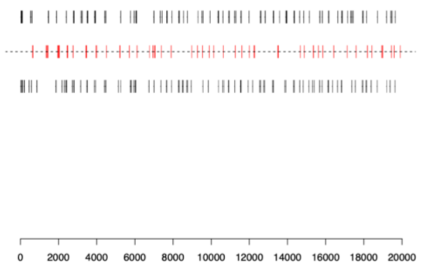

GH010598

Darter

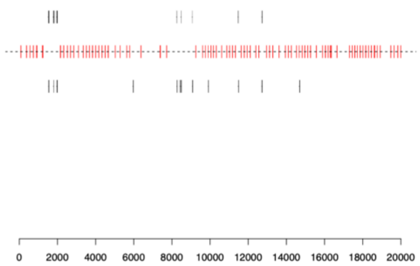

GH010056

Darter

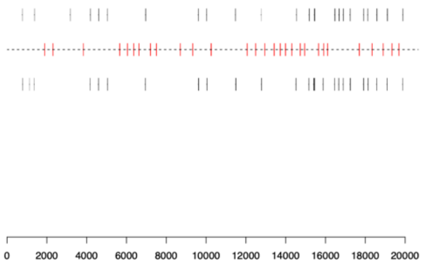

GH010077

Darter

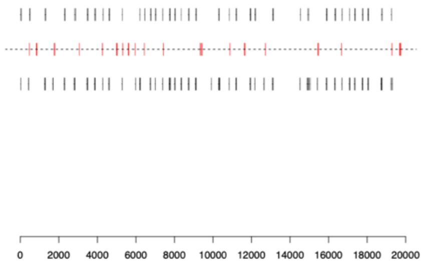

GH010055

Darter

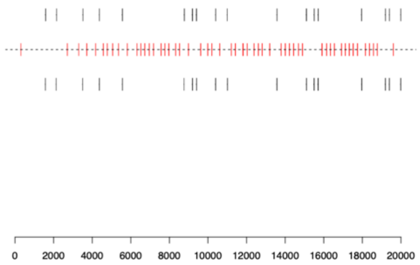

GH010016

Darter

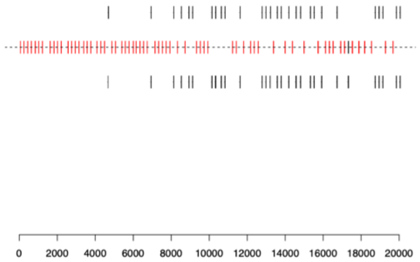

GH010057

Darter

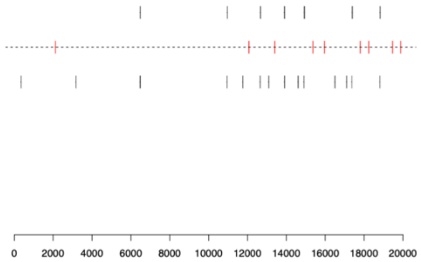

GH010062

Darter

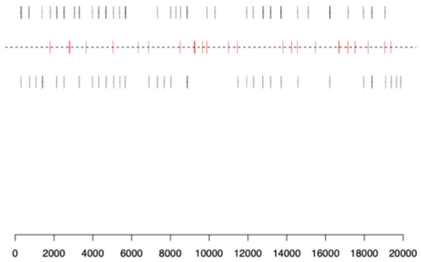

GH010065

Darter

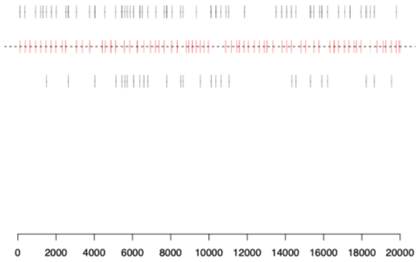

GH010063

Darter

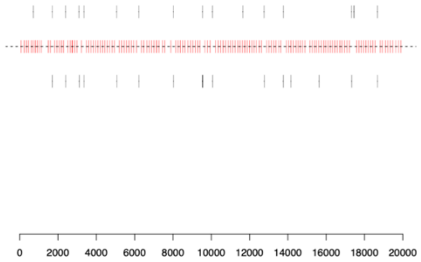

GH010582

Darter

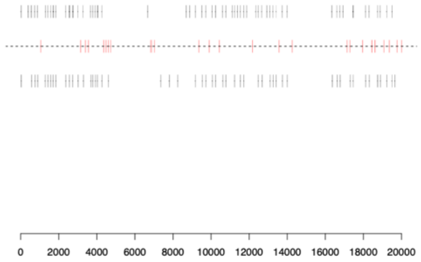

GH010583

Darter

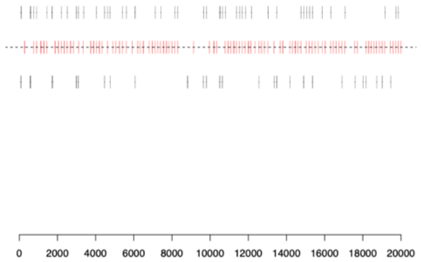

GH010068

Darter

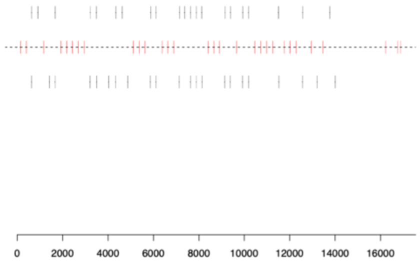

GH010048

Darter

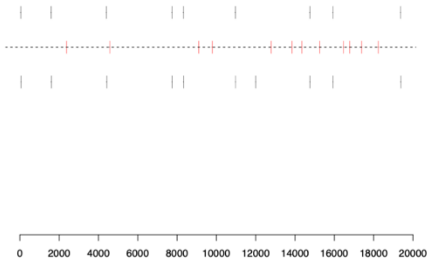

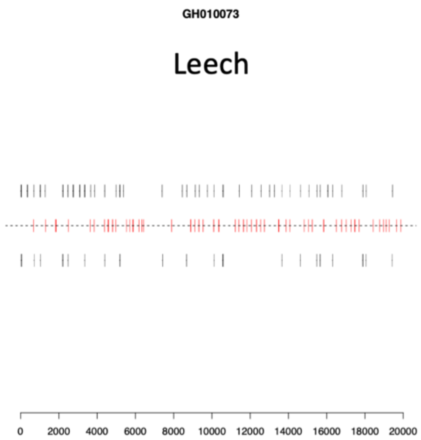
GH010073
Leech
0
2000
4000
6000
8000
10000
12000
14000
16000
18000
20000

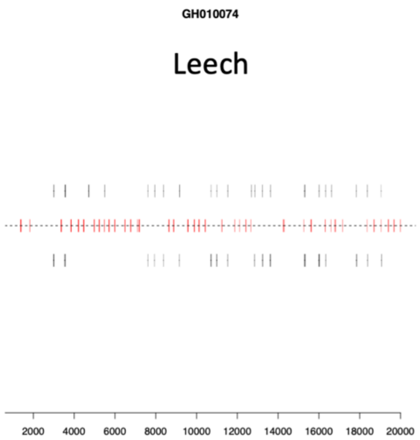
GH010074
Leech
2000
4000
6000
8000
10000
12000
14000
16000
18000
20000

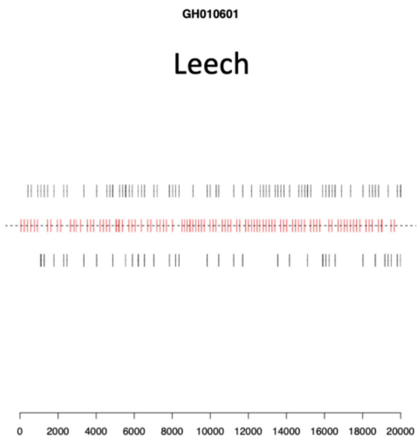
GH010601
Leech
0
2000
4000
6000
8000
10000
12000
14000
16000
18000
20000

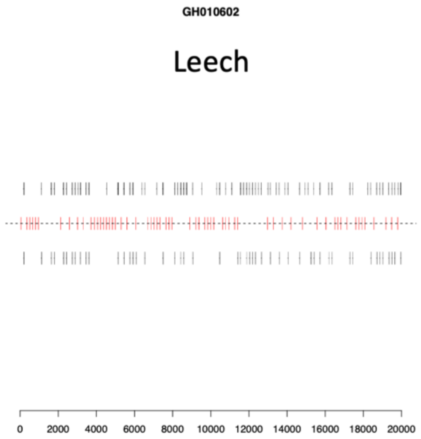
GH010602
Leech
0
2000
4000
6000
8000
10000
12000
14000
16000
18000
20000

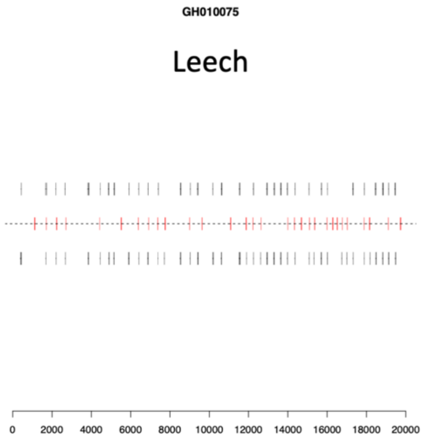
GH010075
Leech
0
2000
4000
6000
8000
10000
12000
14000
16000
18000
20000

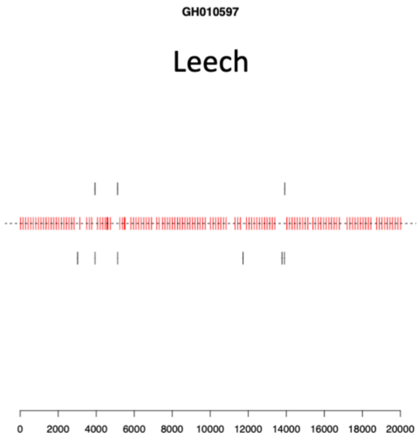
GH010597
Leech
0
2000
4000
6000
8000
10000
12000
14000
16000
18000
20000

GH010595

Leech

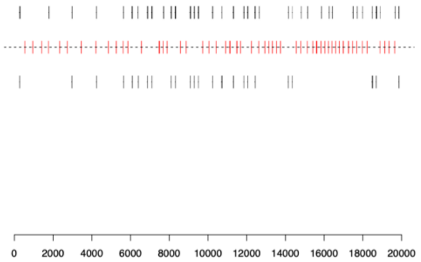

GH010593

Leech

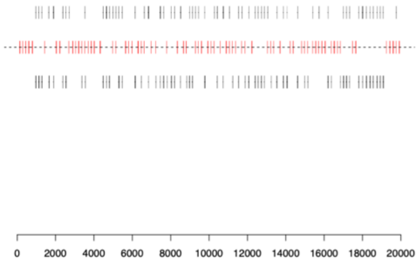

GH010064

Leech

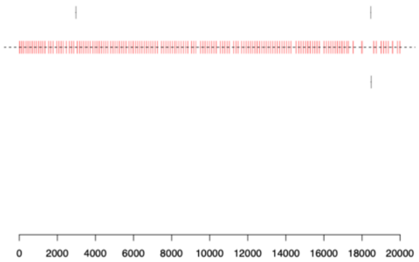

GH010579

Leech

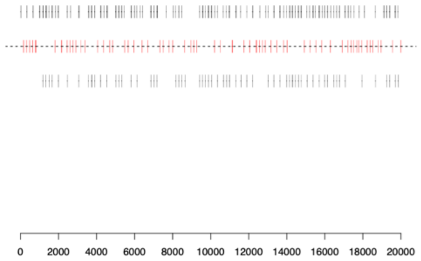

GH010580

Leech

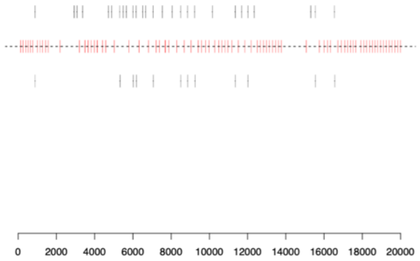

GH010581

Leech

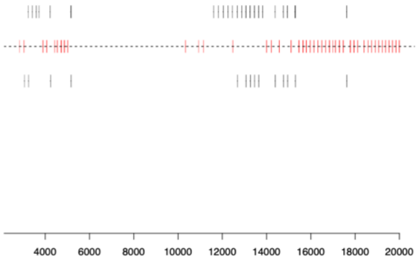

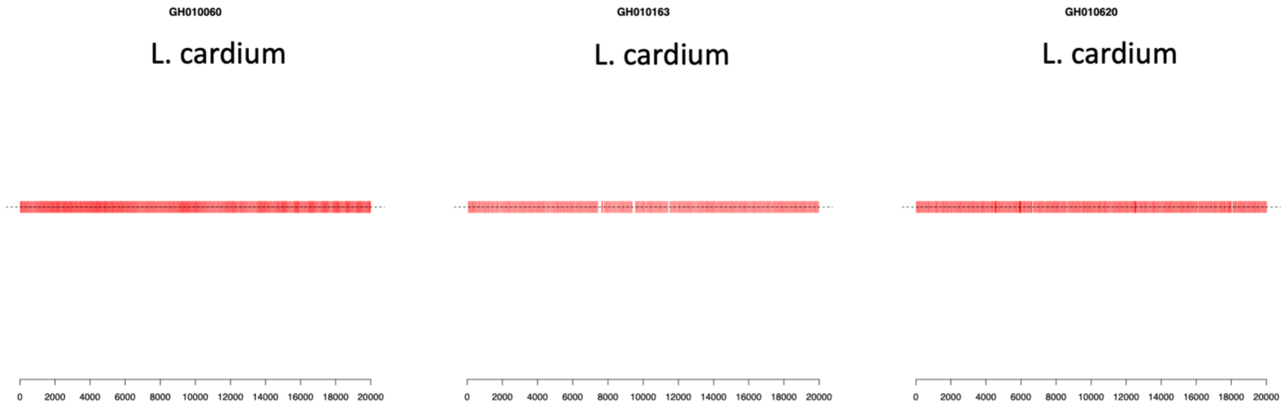

Supplementary Figure 6: Gait analyses for *Lampsilis fasciola* and *Lampsilis cardium* lure behavior. X axis denotes frame number. Videos were taken in Summer of 2018 from Sharon Mills (Fig. 2a) and Hudson Mills (Fig. 2b). Red lines on the center dotted line represent synchronized left/right movements, and black lines above the center line represent left side movements and below represent right side movements. Each graph is labelled as either a darter-like *L. fasciola*, leech-like *L. fasciola,* or *L. cardium.*
